# Which interventions optimize antibiotic prescribing in primary care in England? A survey and Qualitative Comparative Analysis of NHS Integrated Care Boards

**DOI:** 10.1093/jacamr/dlaf244

**Published:** 2025-12-16

**Authors:** Rebecca Knowles, Clare I R Chandler, Stephen O’Neill, Nicholas Mays

**Affiliations:** Department of Health Services Research and Policy, London School of Hygiene & Tropical Medicine, 15-17 Tavistock Place, London WC1H 9SH, UK; Department of Global Health and Development, London School of Hygiene & Tropical Medicine, 15-17 Tavistock Place, London WC1H 9SH, UK; Department of Health Services Research and Policy, London School of Hygiene & Tropical Medicine, 15-17 Tavistock Place, London WC1H 9SH, UK; Department of Health Services Research and Policy, London School of Hygiene & Tropical Medicine, 15-17 Tavistock Place, London WC1H 9SH, UK

## Abstract

**Background:**

Optimizing antibiotic use is a UK Government priority. This study aimed to identify which combinations of interventions are associated with meeting primary care antibiotic prescribing targets in England’s National Health Service, going beyond typical evaluations of individual interventions.

**Methods:**

Data on interventions implemented by Integrated Care Boards (ICBs) in England were collected via an online survey (October 2023 to January 2024). The survey gathered information about 61 interventions covering data monitoring, incentives, governance, staff training, guidance, diagnostics, decision support tools and public awareness-raising activities.

The survey data were linked to ICB-level antibiotic prescribing data, analysed descriptively and through a set-theoretic approach (fuzzy-set Qualitative Comparative Analysis, fsQCA). Clusters of ICBs that used a common set of interventions and met prescribing targets were identified. The average prescribing rates were calculated for each cluster and compared with ICBs that did not implement those interventions.

**Results:**

Fifty-four responses were received from staff at 29 out of 42 ICBs (69%). Locally adapted prescribing guidance was used by all ICBs meeting targets. ICBs that monitored data and used incentives, guidance and/or challenged prescribers on their behaviour had the lowest prescribing. Implementing diagnostics, staff training or public awareness-raising interventions was not associated with lower prescribing.

**Conclusions:**

In a country that has been reducing antibiotic prescribing and implementing numerous antimicrobial stewardship interventions over the last decade, commissioning organizations that met policy targets were using combinations of a limited number of interventions by 2024. National and local efforts could therefore start prioritizing fewer interventions to further reduce prescribing.

## Introduction

Antimicrobial resistance (AMR) is a multicausal public health problem and is one of the top ten global health threats according to the WHO.^1,2^ Antibiotic use in human healthcare settings is a major driver of AMR. In the United Kingdom (UK), 72% of antibiotics are prescribed in primary health care and national targets have been set to reduce antibiotic use across the country.^3^ Although antibiotic prescribing is lower in the UK than many other countries, AMR is an increasing problem and there is variation in antibiotic use across the UK that remains unexplained.^3^

A wide range of strategies to address AMR are being deployed. However, the evidence base for which combinations of these interventions will work best to ensure antibiotics are used appropriately has been insufficient to inform policies and planning.^4^ Our recent systematic review identified over 100 different interventions had been used in England between 2013 and 2022 to optimize antibiotic prescribing in healthcare settings.^5^ These interventions attempt to influence prescribing at different levels of the healthcare system simultaneously: structurally (altering the political, social or economic context), behaviourally (affecting the actions individuals take) and technologically (using devices and tools to influence decisions about a patient’s health and care).^6–8^

The review found that few analyses investigated the combined impacts of interventions, and no studies assessed the impacts of adding new interventions to existing efforts.^5^ It is therefore difficult to tease out the effect of a single intervention from others. It is not known which interventions are most useful when they are used in conjunction with others, which interventions do not add value, and which interventions obstruct progress towards reducing prescribing.

In England, two national organizations (the Department of Health and Social Care alongside NHS England) set the structural policies with the intention that these stimulate the use of behavioural and technological interventions locally via Integrated Care Boards (ICBs, which coordinate health services in their areas), and in Primary Care Networks (PCNs, which are groups of general practices where most antibiotic prescribing occurs) (Box 1).^9^ For example, national prescribing targets are linked to financial incentive schemes, and ICBs can then decide what interventions they will use to reduce antibiotic use to meet targets, whether that is training prescribers, raising public awareness, using decision support tools or creating guidance.

Box 1. The role of Integrated Care Boards (ICBs) and Primary Care Networks (PCNs) in England The NHS Long Term Plan published in 2019 laid out the new direction for structuring, planning and funding health and care services in England. The plan included formalizing groups of GP practices into Primary Care Networks (PCNs) to improve primary care service delivery, and creating Integrated Care Systems (ICS) to plan and fund local services in a more connected way.

***Primary Care Networks***

Across England, there are roughly 1,250 PCNs, each covering on average 50,000 people. Although formalized in the NHS Long Term Plan, many GP practices had been operating in networks before 2019. The aim of PCNs is to improve multidisciplinary service delivery by managing resources across neighbouring GP practices, and delivering a wider range of services which may not be practical to deliver in a single practice.^10,11^

***Integrated Care Boards***

The responsibility of planning and funding services sits with England’s 42 ICSs which were formally established in 2022 as the intermediate tier of the health system, between local and national level. Each ICS has an Integrated Care Board (ICB) and covers between roughly 500,000 to 3 million people. ICBs work with other providers such as local authorities, hospital trusts, and PCNs to plan and commission healthcare services.^9,12^

This study therefore aimed to identify which approaches are used to optimize antibiotic prescribing in primary care in England, which combinations of interventions are implemented by ICBs and which combinations of interventions in primary care are associated with meeting national prescribing targets.

## Methods

This was a mixed methods study that started with a survey to capture information on the interventions being implemented in ICBs. The survey data was linked to routinely collected prescribing data then analysed descriptively and through fuzzy-set qualitative comparative analysis (fsQCA).

QCA was chosen because it can help to bridge the gap between variable-based quantitative analysis and case-oriented qualitative research.^13,14^ It was used as a way of systematically identifying the combinations of interventions (the ‘conditions’) in ICBs (the ‘cases’) that co-occur with levels of antibiotic use that achieve national prescribing targets (the ‘outcome’) using Boolean logic.^15^ Each combination of conditions identified is a ‘solution’, and the conditions in solutions can be either ‘necessary’ (i.e. the condition is present in all cases where the outcome occurs but it may not guarantee the outcome on its own) or ‘sufficient’ (i.e. the condition or combination guarantees the outcome but the outcome may also occur through another causal path).

This approach marks a departure from statistical analyses of antibiotic use interventions as the goal was not to determine the effect size but to use the logic of case-study analysis combined with a mathematical framework based on set-theory to understand the configurations of antibiotic use interventions associated with achieving the antibiotic use targets.

Although the steps used are described linearly below, the QCA analysis was an iterative process where the emerging findings were sense-checked with experts in antimicrobial stewardship (AMS) and used to inform decisions about calibration and interpretation.

### Survey

An online survey was administered to all 42 NHS ICBs, covering all of England. The survey questions were developed with input from NHS Regional AMS Leads, who then distributed the survey by email to ICB staff in their region. Medicines optimization teams were also sent the link directly if their email addresses were publicly available online. The survey was available from 27th October 2023 to 15th January 2024, and respondents received one invitation to participate and up to two reminders to complete the survey.

The survey included questions on the respondent’s role and use or perceived relevance of interventions in primary care implemented over a two-year period (October 2021 to October 2023), aligning with those previously identified in a systematic review. Questions required either ordinal or free-text responses [Table 1, Supplemental Material A (available as Supplementary data at *JAC-AMR* Online)].^5^ A description of each intervention is included in Table S1.

**Table 1.** Interventions and categories included in survey

Intervention category and definition	Interventions included in survey
*Structural*	
Policy, commissioning and incentives (*n* = 6): Interventions involving the planning, prioritizing and purchasing of services to achieve specific goals, e.g. financial incentives	Perceived relevance of Medicines Optimization Opportunities, ICB-led incentives, NHS Oversight Framework, GP Contract, PCN Directed Enhanced Service, National Action Plan
Workforce and governance (*n* = 13): Changes to organizational structures and job roles that are involved in antibiotic prescribing, such as embedding clinical pharmacists in primary care	Time protected for the following roles to spend on AMS activities: Head of Medicines Optimization/Management, Medicines Optimization/Management Pharmacists, PCN pharmacist, Medicines Optimization/Management pharmacy technicians, Clinical Director, other pharmacist, nurse, GP, data and digital staff, admin staff Whether governance structures exist: attend ICS AMS committee, challenge prescribers, in regional AMS group
*Behavioural*	
Guidance and toolkits (*n* = 8): Resources used by healthcare professionals and AMS committees to recommend and inform them of appropriate care and services, such as guidelines for specific infections	Frequency of use of: guidance produced by Royal Colleges, local antibiotic guidelines (which are based on national guidance), NHSE AMR Programme FutureNHS workspace, NICE infection management and antimicrobial prescribing guidance, NICE stewardship guidance (NG15), TARGET toolkit resources, TARGET How to…? guides for recurrent infection, UKHSA summary of antimicrobial prescribing guidance table (managing common infections)
Monitoring and feedback (*n* = 13): Interventions based on the collection, use and communication of data to inform clinicians about patterns in prescribing, such as data platforms, dashboards, and audits	Frequency of use of: ePACT2, FingerTips, OpenPrescribing, GP system, PrescQIPP, NHS Oversight Framework Dashboard, Primary Care Indicators, Model Health System, PharmOutcomes, ICB-initiated audits, Broad spectrum audits, GP or PCN initiated audits, TARGET toolkit audits
Professional engagement and training (*n* = 5): Educational and awareness-raising activities for professionals, e.g. conferences, courses for pharmacists or clinicians	Staff attending: short events or webinars <2 h, half day events, whole-day events, multi-day events, accredited courses
Public awareness (*n* = 7): Strategies to engage and educate people who are not experts or professionals in AMR, such as TV adverts	Frequency of use of: leaflets, posters, internet resources, World Antimicrobial Awareness Week (WAAW), conversations with patients, texts to patients, films
*Technological*	
Prescriber tools and diagnostics (*n* = 8): Devices, strategies and tests used by clinicians at the moment of prescription that can affect decisions about prescriptions, such as diagnostic tests	Frequency of use of: delayed prescribing, clinical prediction scores, digital prompts, digital safety alerts, computerized decision support tools. Diagnostics: urine dip-stick tests, C-reactive protein (CRP) tests, procalcitonin (PCT) tests

The results from the survey were analysed descriptively. Where multiple people from the same ICB responded, the respondent that answered ‘Yes’ to ‘Are you responsible for AMS in your organization’ was used in subsequent analysis. Where an ICB and a representative from a GP practice or ICB both responded for an area, the ICB response was used. The questions on the use or relevance of interventions employed an ordinal scale (highest numbers reflecting the most regular use or perceived relevance of the intervention).

The survey data were linked at the ICB level to antibiotic prescribing data from the NHS Oversight Framework (NOF) prescribing dashboard on NHS England’s AMR Programme FutureNHS Workspace, as well as contextual data on ICB characteristics.^16–20^ Data in the NOF prescribing dashboard is from the NHS Business Services Authority, and contains data based on remuneration of prescriptions dispensed in England.

The monthly average antibiotic use from September 2021 to September 2022 was used as this was the period least likely to be affected by confounding from significant infectious disease events (COVID-19, pandemic-related lockdowns, Group A Streptococcus outbreak between September 2022 and March 2023, and higher rates of flu, pertussis and respiratory syncytial virus in the community due to lower immunity from 2020 to 2022, Figure 1).^3^

**Figure 1.**
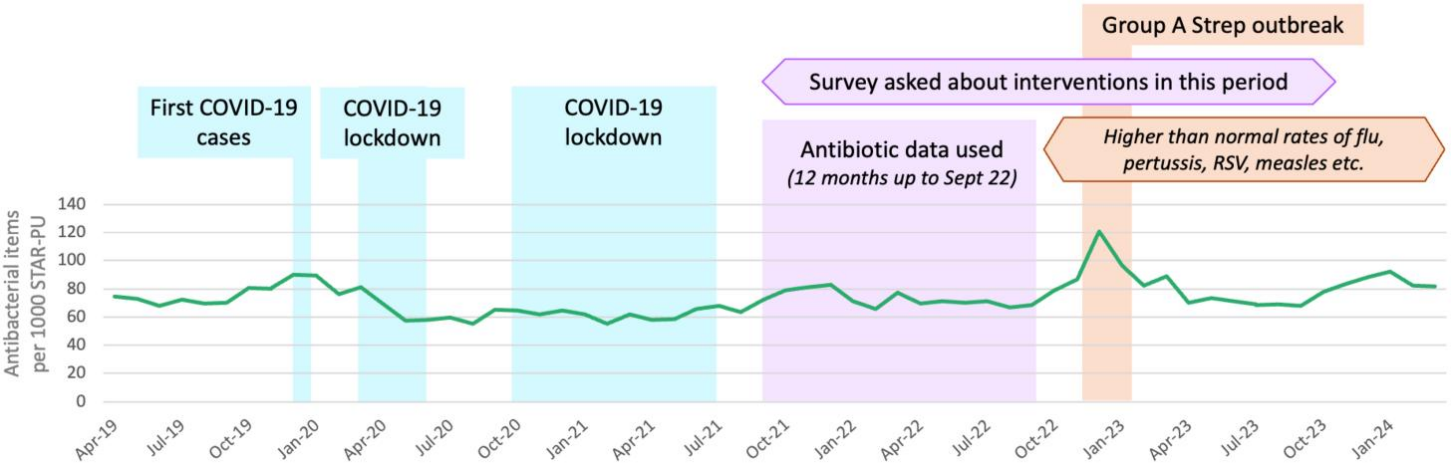
Antibiotic use in primary care in England April 2019 to January 2024. *Before COVID-19, antibiotic use followed clear seasonal patterns (high in winter, low in summer). Lockdowns led to abnormally low antibiotic use, but also subsequent higher infection rates in the community, particularly in children who had lower immunity. Because of this, antibiotic data used in this study is from September 2022 (using a rolling average of the previous 12 months) as this was the period least likely to be affected by confounding. Data from the NHS Oversight Framework (NOF) prescribing dashboard on NHS England’s AMR Programme FutureNHS Workspace* ^*43*^.

Antibiotic use was categorized into three groups aligned with the NOF and the AMR National Action Plan targets: items prescribed per Specific Therapeutic group Age-sex Related Prescribing Unit (STAR-PU) 0.965 to ≤1.161 (the 2014 baseline); 0.871 to ≤0.965 (meeting an interim target); and ≤0.871 (meeting the 2024 prescribing target, reflecting a 25% reduction compared with the 2014 baseline).^21^ In England, STAR-PU is used to account for age and gender profiles that may explain legitimate prescribing variation between GPs, thus enabling a more fair and meaningful comparison between practices.^22,23^

The contextual data on ICB characteristics included factors which have previously been described as affecting antibiotic prescribing:

- Deprivation (Index of Multiple Deprivation)^9^
- Prevalence of comorbidities which have been previously associated with higher antibiotic use in other studies (asthma, diabetes, cancer, chronic obstructive pulmonary disease, chronic kidney disease)^22,23^
- GP workforce (full-time equivalent GPs per 100 000 patients)^16^

As the data was not normally distributed, Wilcoxon rank-sum tests were done to assess whether there were any statistically significant differences between ICBs that responded to the survey and those that did not.

### Fuzzy-set qualitative comparative analysis

#### Background to QCA

QCA was developed by Charles Ragin to study and compare political systems, initially used to understand the causes of political events in different countries where the number of occurrences of a socio-political phenomenon were too few for multivariate statistical techniques such as regression-based analyses.^13^ The method can use binary variables in the conditions and outcomes (either they occurred or they did not occur), called crisp set, or continuous, more granular variables, when conditions and outcomes are on a scale between 1 (representing full membership within a set, which in this case is a group of ICBs) and 0 (representing full non-membership), called fuzzy-set (fsQCA).

QCA identifies necessary and sufficient (combinations of) conditions in solutions. For each solution, the main metrics derived from fsQCA are consistency and coverage:

- Consistency: the degree to which a relationship between a condition or combination of conditions and the outcome comes close to set-theoretic necessity or sufficiency (noting that the consistency score benchmark is higher for determining necessary conditions than it is for sufficient conditions).^14^
- Coverage: the degree to which a cause or causal combination accounts for instances of an outcome.^14^

These metrics are typically presented in a ‘truth table’ which shows them alongside the configurations of conditions co-occurring with the outcome in solutions. The benchmark for a solution to be deemed ‘sufficient’ used in this study was >0.75 consistency.^14^ The benchmark for a condition to be ‘necessary’ for an outcome to occur was 0.9 consistency and 0.5 coverage.^24^

#### Condition calibration and case selection

All ICBs that responded to the survey were included as cases in the fsQCA analysis (Table S2, Step 1). To calibrate the conditions, fuzzy scores between 0 and 1 were assigned based on the extent to which an ICB implemented each intervention (Table 2, Table S2 Step 2, Table S3). For the outcome, scores were assigned based on the degree of attainment of the national policy targets for antibacterial items prescribed per STAR-PU: 0 (reflecting the 2014 baseline of 0.965 to ≤1.61), 0.5 (reflecting the intermediate target of 0.871 to ≤0.965), and 1.0 (reflecting the 2024 target and a 25% reduction from 2014, of ≤0.871) (Table S4).

**Table 2.** Summary of condition calibration

fsQCA score	fsQCA set label	Meaning in this study (example)
1.0	Fully in	An intervention was deemed to be fully implemented (e.g. guidelines were used weekly or more)
0.7	More in than out	An ICB implemented an intervention but not fully (e.g. guidelines were used monthly or every few months)
0.5	Neither in nor out	It was not possible to determine whether the intervention had been implemented (e.g. respondent did not know about implementation)
0.3	More out than in	There was very limited implementation of an intervention (e.g. guidelines were used annually or less)
0.0	Fully out	An intervention was not implemented at all (e.g. when ICBs were aware of a guideline but did not use it or they had not heard of it)

#### fsQCA analysis

The essential components of fsQCA are described here, with a more detailed explanation of the purpose of each step provided in Table S2 and Figure S1. fsQCA software was used for analysis.^25^

The survey gathered information on 61 interventions, a selection of the 109 that had been identified in a previous systematic review.^5^ The systematic review covered both primary and secondary care (whereas the survey only covered primary care interventions), and also spanned a longer time frame (2013–23) than the survey (2021–2023). Therefore, experts at NHS England were consulted on the selection of interventions to include in the survey.

From the 61 interventions, 49 ‘conditions’ were selected and calibrated for the fsQCA with the reduction reflecting the aggregation of some interventions into one condition for the model (Table S2, Steps 2 and 3). First, a ‘necessity analysis’ was done to determine whether any individual interventions always occurred with the outcome (i.e. were necessary, Table S2, Step 4).

As this would still have been too many interventions for a single QCA model to handle, smaller fsQCA models for groups of similar interventions were first investigated to identify the specific interventions likely to be most important, using a similar approach to Avdagic (Table S2, Step 5).^26,27^ The interventions were grouped according to categories identified in a previous systematic review: guidance and toolkits; monitoring and feedback; professional engagement; public awareness; policy, incentives and commissioning; prescriber tools; and workforce and governance.^5^

**Figure 2.**
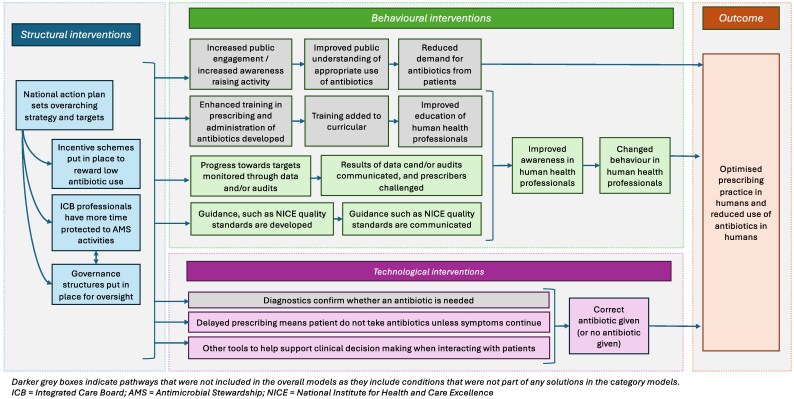
Logic model of interventions. Darker grey boxes indicate pathways that were not included in the overall models as they include conditions that were not part of any solutions in the category model.

The solutions from these smaller models showed which specific (combinations of) interventions were sufficient for an outcome to occur when considering similar interventions, determined on the basis of whether the solution had a consistency score >0.75.^24^ This information was triangulated with literature, survey data, existing logic models from an evaluation of the UK’s AMR Strategy (2013–18, Table S2 Step 6) and whether any individual interventions always occurred with the outcome (i.e. were necessary) to decide which interventions to combine and test in overall fsQCA models (Table 3, Figure 2, Table S2 Step 7).^28,29^ All conditions deemed satisfactory to explore in the overall models were initially included, then conditions were removed if the consistency score was not improved by their presence until there was a minimized solution (the solution with the smallest number of interventions to achieve the consistency score, Table S2 Step 8). The final model then consisted of the simplest combination of interventions for each model, with also the conditions identified as necessary from the necessity analysis (Table S2, Step 9).

**Table 3.** Evidence used in condition selection for overall QCA models

Intervention Category	Summary of findings that have informed condition selection				Conditions included in overall models^b^
	Systematic review findings	Survey	Necessity analysis	Category fsQCA solutions^a^	
Guidance and toolkits	Evidence on uptake and knowledge of specific guidance documents, and effect of TARGET toolkit with professional training	The 3 most used were locally developed, NICE infection management, TARGET.	Local guidance necessary.	Solutions all had NICE infection management and UKHSA guidance	Three interventions: Local guidance, UKHSA guidance, NICE infection management
Policy, incentives and commissioning	Controlled before-after studies that QP (now NOF) was effective at reducing antibiotic use. No evidence for others yet.	Incentives regularly affect ICBs work, but there are several targets and incentives that exist.	No conditions	Solution had ICB incentives, MO, NOF	Three interventions: NOF, ICB-led incentives, MO
Professional engagement and training	Limited evidence of effectiveness (only self-reported knowledge). TARGET webinars and Antibiotic Guardian Campaign might reduce prescribing	Staff mostly attended short events or webinars (<2 hrs).	No conditions	Solution included the absence of attending any training events	None
Public awareness	Limited evidence of effectiveness, not linked to behaviour or prescribing metrics	Leaflets and conversations with patients were most used approach, and public awareness activities did not reduce antibiotic prescribing.	No conditions	No solutions identified	None
Workforce and governance	Limited evidence that changes in roles and governance structures impact prescribing	Medicines optimization pharmacists had most time protected for AMS. Most ICBs participate in ICS AMS Committees and regional AMS groups. Lower prescribing in ICBs that challenged prescribers.	No conditions	Solution had two configurations: (i) time protected for pharmacist technicians and attending the ICS AMS committee, and (ii) time protected for pharmacist and challenging prescribers.	Two combinations of two interventions: (i) Medicines Optimization technician + ICS committee (ii) Challenge prescribers + meds opts pharmacists
Monitoring and feedback	RCTs show that social norms interventions reduce prescribing, but no evidence for specific datasets or audits.	Open Prescribing, PrescQIPP and ePACT2 each used by more than 90% of ICBs. Most ICBs ran their own audits annually, with other audits likely run by GP practices.	No conditions	Solution had ePACT2, Fingertips, Open Prescribing, PrescQIPP. No solutions for audits on their own	Five interventions: ePACT2, Open Prescribing, PrescQIPP, Fingertips, ICB-led audits
Prescriber tools and diagnostics	RCTs show diagnostics can reduce prescribing but financial and operational barriers prevent their use. Evidence is limited for other tools.	Delayed prescribing was most used (with greater use in low-prescribing ICBs).	No conditions	Solution had computerized decision support tools, delayed prescribing, digital prompts, digital safety alerts and text to patients	Four interventions: Computerized decision support tools, delayed prescribing, digital prompts, and text to patients

NAP, national action plan; MO, medicines optimization opportunities; RCT, randomized controlled trial; ICB, Integrated Care Board; AMS, antimicrobial stewardship; QP, quality premium; NICE, National Institute for Health and Care Excellence; TARGET, Treat Antibiotics Responsibly, Guidance, Education and Tools; UKHSA, UK Health Security Agency.

^a^See Table S5 for the solutions from the category models.

^b^Some conditions were subsequently removed in final models as their inclusion did not improve consistency or coverage scores.

Where a solution was deemed sufficient for an outcome to occur, ICBs which had implemented the combination of interventions in the solution were identified as a cluster of cases (implementation was defined as the calibrated fuzzy score for each condition in the solution being ≥0.7). The mean antibiotic prescribing rate (average monthly items prescribing per 1000 population, and per STAR-PU) was calculated for implementing ICBs and compared to non-implementing ICBs, with the difference in means between the two groups assessed.^23^

Other characteristics which have previously been described as affecting antibiotic prescribing were also compared across implementing and non-implementing ICBs for each solution, but, as there were no statistically significant differences in the levels of these characteristics, they were not included as conditions in models (Table S6).

### Impacts of different configurations of interventions at national level

To interpret the results within a broader context, we estimated (with appropriate caution, and assuming the impacts on prescribing are transportable between ICBs) the potential reductions in antibiotic prescribing at a national level were all ICBs to implement each configuration of interventions. The national annual difference in antibiotic items prescribed between implementing and non-implementing ICBs was calculated [difference in antibiotic prescribing per 1000 population × 1000 × 42 (number of ICBs) × 12 (months)] to identify the potential impact if all ICBs implemented each combination of interventions (Table S2, Step 10).

### Contrarian case analysis

Finally, a contrarian case analysis was done to investigate ICBs that did not meet the antibiotic use target. To do this, the same fsQCA models were run but with the outcome of interest set to 0 (i.e. not meeting the target) rather than 1 (meeting the target)^30^ (Table S2, Step 11).

## Results

### Survey results

The survey received 54 responses from 29 out of 42 ICBs (response rate 69%, with responding ICBs covering 72% of the population). Each region in England had at least 50% of its ICBs responding. Most (*n* = 36, 67%) respondents worked in ICBs and were pharmacists (*n* = 31, 57%). The majority described themselves as responsible for AMS (*n* = 42, 78%) and as AMR champions or guardians (*n* = 49, 91%) (Table 4).

**Table 4.** Survey respondent characteristics

Characteristic	Responsible for AMS, *n* (%)		Total, *n* (%)
	Yes	No	
Role			
Medicines optimization pharmacist	16 (89%)	2 (11%)	18 (33%)
Other pharmacist roles	8 (62%)	5 (38%)	13 (24%)
AMS lead	10 (100%)	0 (0%)	10 (19%)
Other roles	5 (83%)	1 (17%)	6 (11%)
Head of medicines optimization	3 (75%)	1 (25%)	4 (7%)
GP	0 (0%)	3 (100%)	3 (6%)
Type of organization			
ICB	31 (86%)	5 (14%)	36 (67%)
PCN	4 (100%)	0 (0%)	4 (7%)
GP practice	2 (50%)	2 (50%)	4 (7%)
Other	9 (90%)	1 (10%)	10 (19%)
Describe themselves as an AMR champion or guardian			
Yes	41 (85%)	8 (15%)	49 (91%)
No	1 (20%)	4 (80%)	5 (9%)

Of the ICBs that responded to the survey, 39% (*n* = 11) met the prescribing target, and prescribed on average 0.88 antibacterial items per STAR-PU (ranging from 0.62 to 1.08 items per STAR-PU) (Table 5). There was clear geographic variation in prescribing, with ICBs within London having the lowest rates whilst ICBs in the North East had the highest. There was no statistically significant differences in characteristics between ICBs that responded to the survey and those that did not (Table 5).

**Table 5.** Characteristics of ICBs

Characteristic	Responded to survey		*P*value
	Yes (*n* = 29)	No (*n* = 13)	
***Size*** ^18^			
Population size *(mean n per ICB)*	1 527 656	1 353 963	0.523
Population size *(total n of all ICBs)*	44 302 026	17 601 521	—
***Comorbidities’ prevalence*** ^19^			
Asthma *(mean %)*	6.79	6.33	0.057
Diabetes *(mean %)*	7.45	7.49	0.838
Chronic obstructive pulmonary disease *(mean %)*	1.94	1.65	0.052
Cancer *(mean %)*	3.68	3.52	0.514
Chronic kidney disease *(mean %)*	4.42	4.39	0.775
***Deprivation*** ^17^			
Proportion in lowest IMD quintile *(mean %)*	18.3	15.1	0.216
***GP workforce*** ^16^			
FTE GPs per weighted 100 000 population *(mean n)*	59.2	58.0	0.438
***Antibiotic use*** ^20^			
Antibacterial items per STAR-PU *(monthly average September 2021-September 2022)*	0.88	0.88	0.663
ICBs that met prescribing target September 2022 *(%)*	37.9	46.2	0.860

The *P* value is from Wilcoxon rank-sum tests performed to test whether ICBs that responded to the survey were different based on key characteristics.

Monitoring and feedback interventions were reported to be the most useful interventions by survey respondents, followed by guidance and toolkits, and then policy and commissioning interventions. It is worth noting that the adoption of interventions will be impacted by prior prescribing and behaviour—ICBs may have implemented interventions in response to high or low-prescribing rates, rather than interventions leading to high or low-prescribing rates.

For *monitoring and feedback interventions*, three data platforms (OpenPrescribing, PrescQIPP and ePACT2) were used by over 90% (*n* = 27) of responding ICBs. In addition, 93% (*n* = 25) of ICBs ran their own audits (usually annually), with 85% (*n* = 23) also conducting audits specifically on broad spectrum prescribing. Seventy percent (*n* = 7) of ICBs that met the target on 0.871 antibacterial items per STAR-PU challenged prescribers on the prescribing patterns, compared to 473% (*n* = 10) of ICBs that did not meet the target.

For *guidance and toolkit interventions*, the National Institute for Health and Care Excellence (NICE) Infection Management was used by all ICBs (*n* = 29) at least every few months. Locally adapted guidelines (which are often based on national guidelines), the TARGET toolkit and the UK Health Security Agency’s (UKHSA) summary table were used by over 90% of ICBs too (*n* = 27). The guidelines used regularly tended to provide specific advice on prescribing based on infections rather than broader guidance on AMS. For example, 79% (*n* = 23) of ICBs used NICE Infection Management guidance at least monthly, whilst 62% (*n* = 18) of ICBs used NICE Stewardship guidance every few months or less. Eighty percent (*n* = 8) of ICBs that met the prescribing target used between three to five different resources, whereas most of those not meeting the target used six to eight different resources.

For *policy, commissioning and incentive interventions*, there were numerous targets and incentives for primary care prescribing which aimed to reduce antibiotic course length [including NAP Targets and Medicines Optimization Opportunities (MO) target 14], reduce the total volume of prescribing (NOF44a), reduce the proportion of broad spectrum prescribing (NOF44b) and increase AMS activities (GP Contract, PCN Directed Enhanced Service). MO was perceived to be the most relevant incentive, but there was no difference in perceived relevance of the MO or NOF targets and antibiotic use outcomes. Ninety percent (*n* = 26) of ICBs felt that the NAP was relevant for their work.

For *professional engagement and training*, 92% (*n* = 23) of responding ICBs had staff attending short events or webinars, with 48% (*n* = 11) attending half- or whole-day events. One respondent said ‘Short events are helpful to keep up to date in a fast-moving world. Longer face-to-face events are equally beneficial due to networking opportunities’. Seventy percent (*n* = 7) of ICBs that met the prescribing targets had at least one member of staff attend training events (mostly short events or webinars), compared to 89% of ICBs that did not meet the target.

For *prescriber tools and diagnostics*, some ICBs did not feel able to respond to questions about their use and implementation, stating that this was at the discretion of clinical teams. Information on the use of diagnostics was only collected from 19 ICBs and for prescriber tools from 21 ICBs. Where ICBs did respond, delayed (or back-up) prescribing was most used, being implemented by 76% (*n* = 18) of ICBs. Eighty-three percent (*n* = 5) of ICBs that met the target encouraged the use of delayed prescribing at least weekly, compared to 73% (*n* = 11) of ICBs that did not meet the target. Computerized decision support tools were used by 62% of ICBs (*n* = 13). Some (38%, *n* = 11) ICBs also used diagnostics tests to improve prescribing, such as tests for urinary tract infections (UTIs), C-reactive protein (CRP) and procalcitonin (PCT) in certain situations. One respondent said ‘we trialled CRP for RTI (respiratory tract infections) in primary care some years ago… some practices reduced antibiotic prescribing, some increased. There was no funding available when the study ended and GP practices were not prepared to fund themselves’.

For *public awareness interventions*, ICBs used up to seven different approaches to engage the public. These interventions were used regularly with 61% (*n* = 14) of ICBs encouraging clinicians to have conversations with patients, 64% (*n* = 16) using leaflets and 38% (*n* = 9) directing patients to internet resources on a weekly basis or more. Sixty percent (*n* = 6) of ICBs that met the target implemented any combination of public awareness interventions, compared to 79% (*n* = 15) of ICBs that did not meet the target.

For *workforce and governance interventions*, Medicines Optimization Pharmacists were the most likely role to have time protected, with 41% (*n* = 11) of ICBs having the equivalent of one day protected per week for that role. Ninety-three percent (*n* = 27) of ICBs had a representative attend the ICS AMS Committee and 66% (*n* = 19) participated in regional AMS groups. Thirty-three percent (*n* = 3) of ICBs that met the prescribing target had time protected for Medicines Optimization Pharmacists to spend on AMS, compared to 61% (*n* = 11) of ICBs that did not meet the target.

### Results from fsQCA

The local adaptation of guidance was the only necessary condition (coverage = 0.56, consistency = 0.90), used by 93% (*n* = 27) of responding ICBs (noting that local documents are based on national guidance, rather than being guidance that has been made specifically for each local context). This means that all ICBs which met the target implemented locally adaptation guidance (*n* = 10), but not all those that implemented this guidance met the target (*n* = 17).

#### Interventions selected within each category

In addition to the local adaptation of guidance (the only necessary condition), each category of interventions had a selection of interventions which were identified as being sufficient.

The (combinations) of interventions within each category that when present, the outcome was also present (Table 3, Table S5) were:

- Guidance and toolkits (three interventions were sufficient when implemented as a combination, out of eight implemented): using UKHSA guidance, NICE Infection Management and locally adapted guidance
- Monitoring and feedback (five interventions were sufficient when implemented as a combination, out of nine implemented): using the data platforms ePACT2, OpenPrescribing, PrescQIPP, and Fingertips, and doing ICB-led audits
- Policy, incentives and commissioning four interventions were sufficient when implemented as a combination, out of six implemented): high perceived relevance of the NOF, ICB-led incentives, MO and the UK’s AMR NAP
- Prescriber tools and diagnostics (five interventions were sufficient when implemented as a combination, out of eight implemented): using clinical decision support tools, delayed prescribing, digital prompts, digital safety alerts and texting patients
- Workforce and governance (four interventions were sufficient when implemented as a combination, out of six implemented): having time protected for medicines optimization technician or pharmacists to spend on AMS activities, having a representative on the ICS AMS committee, and challenging prescribers on their prescribing behaviours.

By 2024, there were also some interventions within each category that were not (or no longer) sufficient or necessary for the outcome to occur. This includes any combination of the seven public awareness interventions implemented or the five professional engagement and training interventions. Combinations of the three diagnostic tests included in the survey were also not found to be necessary or sufficient (C-reactive protein, dip-stick tests for UTIs, and PCT).

#### Combining different types of interventions

**Figure 3.**
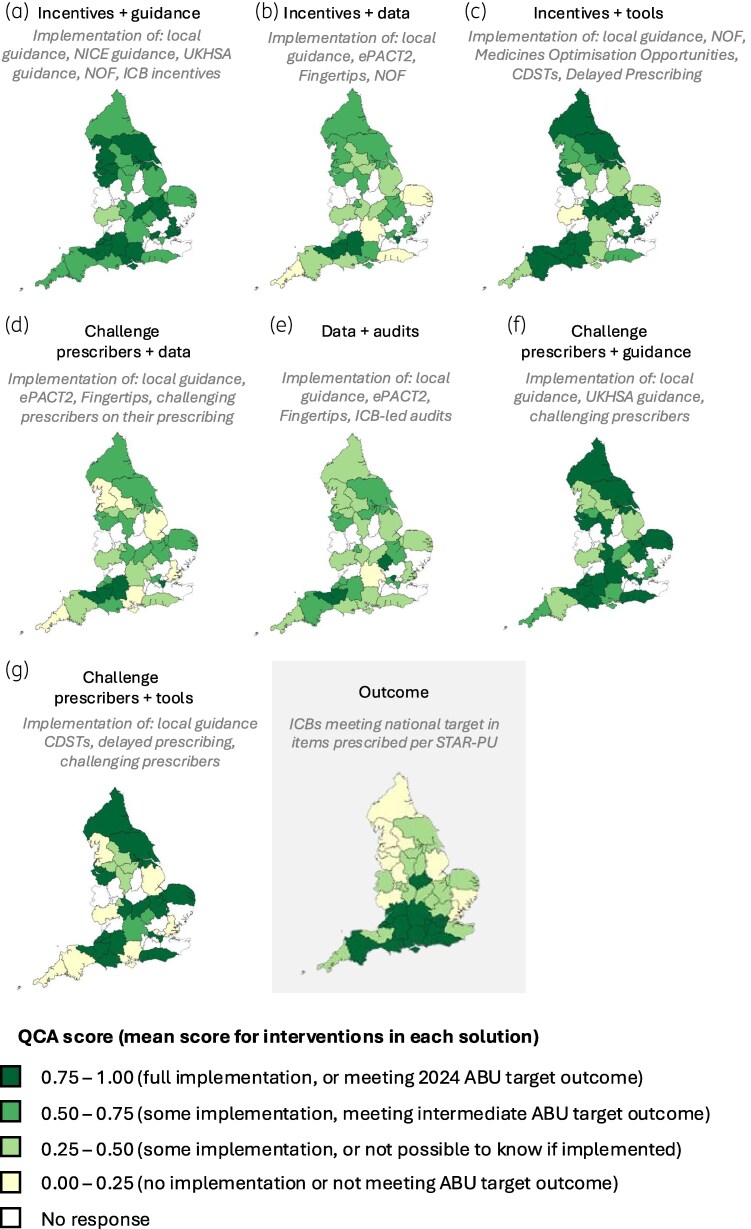
Implementation of antibiotic use interventions in primary care in England.

The sufficient interventions in each category were then combined with those in other categories, based on the seven pathways hypothesized from a logic model to form the ‘overall models’ (Figure 2). After minimizing these overall models to find the simplest combination of solutions, the following configurations of interventions were sufficient for ICBs to meet prescribing targets when implemented with the local adaptation of guidance (Table 6, Figure 3):

- Incentives (NOF, ICB-led) and guidance (NICE Infection Management, UKHSA guidance)
- Incentives (NOF) and data (ePACT2, Fingertips)
- Incentives (NOF, MO) and prescriber tools (Delayed prescribing, computerized decision support tools)
- Challenging prescribers and data (ePACT2, Fingertips)
- Data (ePACT2, Fingertips) and audits (ICB-led)
- Challenging prescribers and guidance (Local guidance, UKHSA guidance)
- Challenging prescribers and prescriber tools (Delayed prescribing, computerized decision support tools)

**Table 6.** Combinations of interventions that were sufficient for ICBs to meet antibiotic use targets

	Configurations		fsQCA metrics			Antibiotic items prescribed per 1000 patients (mean)			Antibiotic items prescribed per STAR-PU (mean)		
	Summary (in additional to local guidance)	Conditions	Coverage	Consistency	No. ICBs	Implementing ICBs	Non-implementing ICBs	Difference	Implementing ICBs	Non-implementing ICBs	Difference
A	Incentives + guidance	Local guidance* NICE guidance* UKHSA guidance *NOF*ICB Incentives	0.29	0.82	20	38.0	42.3	−4.3	0.87	0.91	−0.04
B	Incentives + data	Local guidance* ePACT2* Fingertips*NOF	0.41	0.91	13	36.0	42.3	−6.4	0.83	0.93	−0.10
C	Incentives + prescriber tools	Local guidance* NOF*MO* CDSTs*Delayed prescribing	0.28	0.89	9	40.5	39.0	1.5	0.92	0.87	0.05
D	Challenge prescribers + data	Local guidance* ePACT2*Fingertips* Prescribers challenged	0.34	0.87	9	36.1	41.0	−4.9	0.82	0.91	−0.10
E	Data + audits	Local guidance* ePACT2*Fingertips *ICB audit	0.46	0.89	11	35.5	41.9	−6.4	0.83	0.94	−0.11
F	Challenge prescribers + guidance	Local guidance* UKHSA guidance* Prescribers challenged	0.22	0.89	15	38.0	41.0	−3.0	0.85	0.92	−0.07
G	Challenge prescribers + tools	Local guidance* CDST*Delayed prescribing* Prescribers challenged	0.29	0.92	7	40.1	39.3	0.8	0.89	0.88	0.01

CDST, computerized decision support tools; NOF, National Oversight Framework; MO, medicine optimization opportunities, ICB, integrated care board; fsQCA, fuzzy-set qualitative comparative analysis; STAR-PU, specific therapeutic group age-sex related prescribing Uni; ePACT2, an NHS-run data platform.

Antibiotic items prescribed per 1000 patients and per STAR-PU is the monthly average from September 2021–22.

Time protected for staff to spend on AMS activities did not improve or decrease the consistency scores, so those conditions were not included in final models.

ICBs that monitored data regularly with either audits or national incentive mechanisms had lower prescribing than other ICBs (Table 6). ICBs which used ePACT2 and Fingertips data platforms at least monthly whilst also reporting that NOF incentives affected their work at least weekly, prescribed on average 6.4 fewer antibiotic items per 1000 patients each month than ICBs that did not implement those interventions (Solution B). Similarly, ICBs that used those datasets whilst also either challenging prescribers on their prescribing behaviour or conducting ICB-led audits, prescribed on average 4.9 and 6.4 fewer antibiotic items per 1000 patients each month respectively than non-implementing ICBs (Solutions D and E).

ICBs that used guidance also had lower prescribing than non-implementing ICBs. Only two ICBs did not use local guidance (neither of which met the NOF antibiotic use target). ICBs which reported that incentives affected their work weekly or more, and used NICE Infection Management and UKHSA guidance at least every few months, prescribed on average 4.3 fewer antibiotic items per 1000 patients each month than ICBs that did not implement those interventions (Solution A). ICBs which used those guidance resources whilst also challenging prescribers prescribed on average 3.0 fewer antibiotic items per 1000 patients each month than ICBs that did not implement those interventions (Solution F).

The two pathways that included prescriber tools had marginally higher prescribing in the ICBs that used them. ICBs that used clinical decision support tools and delayed prescribing alongside incentive schemes (NOF and MO) at least weekly, prescribed on average 1.5 antibiotic items more per 1000 patients each month than other ICBs (Solution C). Similarly, ICBs that used those tools whilst also challenging prescribers on their prescribing practices used 0.8 antibiotic items more per 1000 patients each month than other ICBs (Solution G).

There was no noticeable difference in the characteristics (deprivation, comorbidities, population size, and GP workforce) of the ICBs that implemented different combinations of interventions (Table S6).

### Impacts of different configurations of interventions at national level

**Figure 4.**
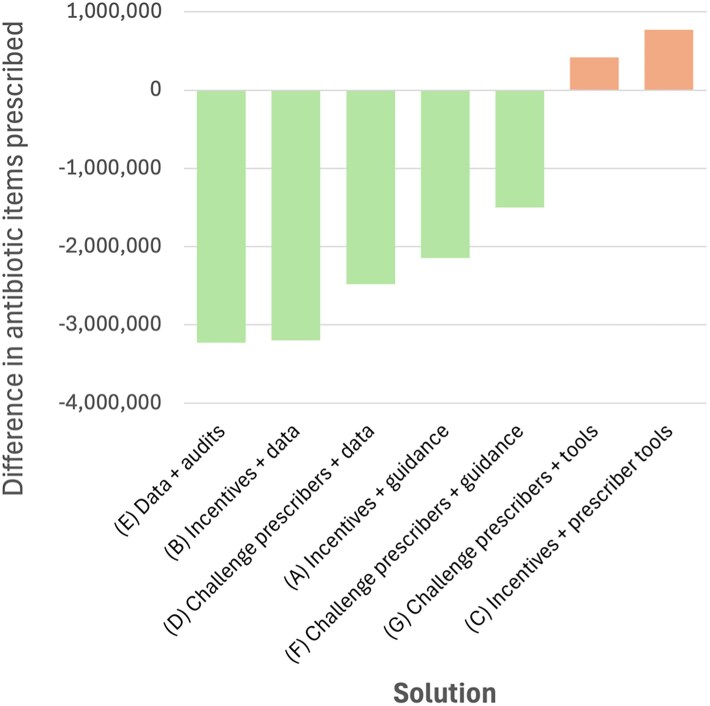
Potential difference in antibiotic prescribing if all ICBs in England implemented each configuration of interventions.

Taking the difference in prescribing between implementing and non-implementing ICBs, it was possible to identify the potential impact on antibiotic prescribing if all ICBs in England implemented each configuration, compared with a situation in which no ICBs implemented them (Figure 4). These calculations are generalizations and operate under the assumption that ICBs have the same context, e.g. same incidence of infectious diseases in their populations, comparable population demographics, same resources to implement interventions, and prescribers who behave in the same way in response to interventions.

Compared to if no ICB implemented the interventions identified, if all ICBs used data systems (ePACT2 and Fingertips) regularly with audits or incentive mechanisms, there could be approximately 3 million fewer antibiotic items prescribed in England in a year, approximately 10% of the national total prescribing: 3 225 096 fewer antibiotic items for data plus audits (Solution E); and 3 197 880 fewer antibiotics for data plus incentives (Solution C).

Three other configurations showed that implementing combinations of interventions could see between 1.5 and 2.5 million fewer antibiotic items prescribed in a year than if no ICB had implemented the solutions: using data and challenging prescribers (Solution D) could see 2 481 192 fewer antibiotics prescribed; using incentives and guidance (Solution A) could see 2 148 048 fewer; and challenging prescribers and using guidance could see 1 498 986 fewer.

The two configurations that produced marginal differences in prescribing could generate a small increase in antibiotic use, if those combinations of interventions were used. These configurations were some prescriber tools with either challenging prescribers (Solution G) or incentives (Solution C) which could see 420 840 and 774 144 more antibiotics used, respectively.

### Contrarian case analysis

The contrarian case analysis showed that some ICBs did not meet the target whilst either implementing local guidance (Solution F), some incentives (Solution C), or delayed prescribing (Solution G) (Table S7). These findings align with the necessity analysis (given all ICBs that met the target implemented local guidance, but not all ICBs that met the target implemented local guidance), and the calculations in antibiotic prescribing across the configurations, where Solutions G and C had higher antibiotic use that the other solutions given not all ICBs that implemented those configurations met the target.

## Discussion

A range of approaches is currently implemented to optimize antibiotic use in human healthcare settings, yet research has largely assessed the value of each intervention in isolation. This study demonstrates that some combinations of interventions frequently occur in primary care settings that meet national antibiotic prescribing targets, but that by 2024, there are also interventions which do not appear to add further benefit to AMS programmes. As the UK Government has announced ambitions to reduce antibiotic prescribing by a further 5% by 2029, all ICBs should be assessing their portfolio of interventions and consider prioritizing the combinations of interventions most likely to be associated with lowest prescribing rates which includes those that monitor prescribing, challenge prescribers on their prescribing patterns, have financial incentives, include audits and/or provide guidance.^31^ Using tools which are intended to support clinical decision making (including diagnostics) or raising public awareness did not appear to contribute to meeting prescribing targets at this point in time, even when used in combination with other interventions.

Antibiotic prescribing has been declining in England since 2013, albeit with a slight increase in 2022 to 2023.^3^ In this context, interventions that monitored prescribing and provided feedback to clinicians have the potential to reduce antibiotic use the most compared to other types of interventions. The combination of two complementary datasets was particularly useful: ePACT2 which provides data on individual prescription items, patient-level detail and high-level summaries through dashboards or reports; and Fingertips which shows trends at the national level for specific indicators and targets.^32,33^ Importantly, the data must be fed back to clinicians to influence their behaviour or linked with financial incentives that reward healthcare settings for meeting prescribing targets, as on their own, data platforms are not enough.^34^

Local adaptation of guidance was the only intervention deemed necessary to support ICBs in meeting prescribing targets, given all 10 of the ICBs that met the target used such guidance. It is worth noting that local documents are largely based on national guidance (rather than the creation of localized guidance based on local context), including those set by UKHSA and NICE, and there were greater potential improvements if guidance resources were used with either incentives or challenging prescribers. Both the UKHSA summary of antimicrobial prescribing guidance (managing common infections) and NICE Infection Management provide infection-specific guidance related to the indications where an antibiotic is required or not. Other resources that were not part of successful configurations (such as NICE Stewardship guidance) were more generic, focusing on the considerations that organizations should give when developing their own AMS programmes.^35–37^

Prescriber tools are designed to help clinicians make decisions at the point of prescription, but no configurations of these tools with (or without) other interventions enabled ICBs to reduce antibiotic use, including tests (CRP, dip-stick or PCT). However, many survey respondents commented that using diagnostics was at the discretion of clinicians rather than the responsibility of service commissioners, and were therefore unsure how often tests were used in practice, so research would be needed to determine the use of diagnostic tests by clinicians in primary care practice. This analysis also reflects the current situation where they are not widely available or used in primary care, but future point-of-care tests and other technologies might have more effect if and when they are available on a larger scale.

Furthermore, computerized decision support tools should provide the prescriber with easy and rapid access to information but it did not appear to add additional benefit to ICBs in meeting antibiotic use targets, even when used with monitoring of prescribing and providing incentives. This may be because decision support tools can be used to help prescribers provide the most appropriate antibiotic for a particular indication, but not necessarily reducing the quantity of antibiotic use.

### Comparison to other studies

Most studies investigating how to optimize antibiotic prescribing have focused on individual interventions despite different combinations of interventions being used in practice. Nevertheless, this study aligns with findings from our earlier systematic review that structural interventions including incentive schemes could have large impacts on prescribing, but it goes further to show that they are likely to be most beneficial when used with data to monitor prescribing or guidance to provide syndrome-specific prescribing advice.^5,38^

Previous studies have shown that guidance was more likely to be effective if implemented alongside staff training, but that was not supported by this research as we found no added benefit of staff attending training events.^39,40^ Data platforms and audits have been studied qualitatively, showing that they prompt staff to identify patterns in over-prescribing and actions to address this.^41^ It has not been possible previously to identify whether using data platforms had a quantitative impact on prescribing, on their own or compared to other interventions. However, earlier evidence has shown that ‘social norms tactics’ (which rely on rules that are understood by members of a community to influence behaviour) have been effective, and it is likely that providing feedback to prescribers or challenging them works in a similar way.^42^

The finding that some interventions were not consistently used in ICBs that met prescribing targets is largely consistent with other literature. Diagnostic tests, for example, can be effective in controlled research settings but tend not to be useful when clinical teams try to implement them in routine practice due to operational barriers (e.g. lack of funding, sharing machines amongst GP practices, etc.).^43^ Furthermore, raising awareness of AMR in the public and professionals has not yet been shown to be effective, as this tends to reach those who are already engaged in the area.^44^

### Strengths and limitations

This was a cross-sectional study aiming to take a high-level view of AMS, therefore the results should be interpreted with care. This analysis represents a snapshot in time in a country that has implemented over 100 interventions in the last decade and which has been reducing its overall antibiotic prescribing.^5^ Therefore, interventions that did not appear to contribute to meeting prescribing targets in this study may have had a greater impact at a different time or in a different setting (such as when England first started implementing AMS interventions and when there were higher prescribing rates).

The main strength and added value of this study is that it investigated combinations of interventions, rather than looking at the impact of single interventions. The data analysed were based on a survey that had a reasonable response rate (69% of ICBs, covering 72% of the population), with good representation across every region in England, and the responses were from those best informed on what was being implemented in each ICB area.

The study was limited by the self-reported survey data, and respondents could have reported activities that they deemed to be more desirable (e.g. showing that they were using more interventions and putting more effort into AMS). The specific timing of when interventions were implemented is unknown, therefore it was not possible to say if ICBs were implementing interventions in response to high prescribing (and there could be a delay before effects are seen), or if interventions were followed by reductions in prescribing.^37^

Furthermore, the scope of this research was limited to assessing where there was low antibiotic use, under the assumption that lower prescribing is better. It was beyond the scope of this study and of the data to investigate whether reducing prescribing resulted in any unintended consequences, however other studies suggest that lower prescribing does not always have negative consequences (such as hospitalizations) in England.^45^

### Conclusion

England has been reducing antibiotic prescribing over the last decade, using a plethora of interventions across the country over this period. Our analysis shows that the interventions used most frequently in commissioning organizations that met primary care antibiotic use targets were nationally-set and locally adapted guidance, as well as data platforms linked with incentive mechanisms and activities challenging prescribers on their behaviour. This suggests that national and local efforts could focus on a smaller number of interventions and still achieve impact.

fsQCA can be useful for assessing which configurations of interventions could be associated or not with desired policy outcomes, moving away from assessing interventions singly. Despite its limitations, is a useful analytical approach when faced with a small number of cases of a phenomenon as with the ICBs in this study.

## Supplementary Material

dlaf244_Supplementary_Data

## Supplementary data

Figure S1 and Tables S1 to S7 are available as Supplementary data at *JAC-AMR* Online.
