# Supplementary material for: Which interventions optimize antibiotic prescribing in primary care in England? A survey and Qualitative Comparative Analysis of NHS Integrated Care Boards
Source: JAC Antimicrob Resist. 2025 Dec 16;7(6):dlaf244. doi: 10.1093/jacamr/dlaf244 (PMC12706466; doi:10.1093/jacamr/dlaf244)
Supplement: dlaf244_Supplementary_Data [file dlaf244_supplementary_data.docx]

# What interventions optimise antibiotic prescribing in primary care in England? A survey to NHS Integrated Care Boards and qualitative comparative analysis

SUPPLEMENTAL MATERIAL

Contents

[A - Survey questions 2](#_Toc215064974)

[B – Interventions used in primary care in England 17](#_Toc215064975)

[Table S1: Description of interventions asked about in the survey 17](#_Toc215064976)

[C – fsQCA methods 24](#_Toc215064977)

[Table S2: Stages of fsQCA 24](#_Toc215064978)

[Figure S1: Stages of fsQCA 28](#_Toc215064979)

[Table S3: Condition calibration 29](#_Toc215064980)

[Table S4: Outcome calibration 30](#_Toc215064981)

[D – Category fsQCA results 31](#_Toc215064982)

[Table S5: Effective solutions identified in category-specific fsQCA (‘truth table’) 31](#_Toc215064983)

[E - Characteristics of ICBs 32](#_Toc215064984)

[Table S6: Characteristics of ICBs that implemented different combinations of interventions 32](#_Toc215064985)

[F – Contrarian case analysis 33](#_Toc215064986)

[Table S7: Analysis results when the outcome of interest is not meeting the target 33](#_Toc215064987)

[G - References 34](#_Toc215064988)

## A - Survey questions

Optimising antibiotic prescribing in primary care in England

**The London School of Hygiene and Tropical Medicine (LSHTM) and NHS England Regional AMS Leads would like to invite you to complete a survey on antimicrobial prescribing interventions in England.**

**What is the purpose of the study?**

LSHTM, with support of the NHS England Regional Antimicrobial Stewardships Leads, is conducting research into the interventions being used to optimise antimicrobial prescribing in England.

There are numerous approaches being used to encourage appropriate antibiotic use, including national strategies, public campaigns, educational programmes, new targets, and data monitoring systems. This research study aims to understand which interventions are being prioritised locally, regionally and see whether any variation in intervention uptake contributes to differences in prescribing.

**Why have I been asked to take part?**

We are asking people from a range of backgrounds who influence prescribing in some way to complete the survey. This includes people involved in the design or implementation of antimicrobial stewardship policies, strategies and interventions, in clinical, pharmacist and commissioning roles.

**What are the possible benefits and risks?**

This study will help us understand what interventions are being used with the results aiming to inform future policies and decisions about antimicrobial resistance in the UK. We can use the information to influence decisions being made by policy and commissioning organisations in the future. This is a low-risk study as we are not asking about any personal or sensitive topics.

**What if something goes wrong?**

If you have a concern about any aspect of this study, you should ask to speak to the researchers who will do their best to answer your questions. If you remain unhappy and wish to complain formally, you can do this by contacting rgio@lshtm.ac.uk or +44 (0) 20 7927 2626.

**Can I change my mind about taking part?**

Yes. You can withdraw from the study at any time, but we may need to use the data collected from you up to your withdrawal.

**What will happen to information collected about me?**

LSHTM is the sponsor for the research and has full responsibility for the project including the collection, storage and analysis of your data, and will act as the Data Controller for the study. This means that the research team, on behalf of LSHTM, will look after your data properly. All the information we collect during the research will be kept confidential and there are strict laws which safeguard your privacy at every stage. To follow the United Kingdom’s data protection regulations, we must inform you of how we will use and store your personal data.

LSHTM uses personally identifiable information to conduct research to improve health, care and services. We have to ensure that it is in the public interest when we use personally identifiable information from people who have agreed to take part in research. This means that when you agree to take part in a research study, we will only use information collected from you to conduct and analyse the research study.

Only the study staff and authorities who check that the study is being carried out properly will be allowed to look at information about you. Information will include your name, contact details and organisation. Your personal details, meaning your name and other identifiable information, will be kept safely in a different place from the other study information and will be stored securely on the LSHTM server.

**Where can you find out more about how your information is used?**

You can find out more about how we use your information at https://www.lshtm.ac.uk/files/research-participant-privacy-notice.pdf or by asking one of the research team by sending an email to DPO@lshtm.ac.uk

**What will happen to the results of this study?**

The study results will be published in a peer-reviewed journal so that others can learn from them. Your personal information will not be included in the study report.

**Who has reviewed this study?**

This study has been reviewed and given favourable opinion by the LSHTM Research Ethics Committee and NHS HRA ethics committee. This project is funded by the Economic and Social Research Council.

**Joining the study is entirely up to you, and if you would like to talk to someone about the study, please contact Rebecca Knowles (Rebecca.Knowles@lshtm.ac.uk ).**

*By completing the survey and providing your email address, you will be eligible for the prize draw to win one of two £100 Amazon vouchers.*

1. Please tick each box to take part in the study: *(Required, tick both)*

- I confirm that I have read and understood the information above for this study
- I agree to taking part in this study

We may contact you after the survey if:

- You have been entered into the prize draw for a £100 Amazon voucher
- To share findings from the survey with you
- To ask whether you would like to take part in a 60minute interview about antimicrobial stewardship interventions with Rebecca Knowles, a Research Degree student at LSHTM

1. Please confirm whether you are happy to be contacted after the survey. *(Required, tick one*)

*Please note, if you would like to be entered into the prize draw for a £100 Amazon voucher, you must select 'I am happy to be contacted'*

- I would like to enter the prize draw and am happy to be contacted
- I do not want to be contacted or entered into the prize draw

Contact details

1. Please add your full name here: *(Required)*

|  |
| --- |

1. Please add your email address here: *(Required)*

|  |
| --- |

1. I would like to be contacted after the survey: *(Tick all that apply)*

- For the findings of the survey to be shared with me
- To take part in an interview on antimicrobial stewardship interventions with Rebecca Knowles, a Research Degree student at LSHTM

Your organisation

1. What type of organisation do you primarily work in? *(Required, tick one)*

- Integrated Care Board or sub-ICB
- Primary Care Network
- GP practice
- Other

If you selected other, please give details:

|  |
| --- |

1. If your work for an ICB, who is your employer? *(Required, tick one)*

- NHS Bath And North East Somerset, Swindon And Wiltshire ICB
- NHS Bedfordshire, Luton And Milton Keynes ICB
- NHS Birmingham And Solihull ICB
- NHS Black Country ICB
- NHS Bristol, North Somerset And South Gloucestershire ICB
- NHS Buckinghamshire, Oxfordshire And Berkshire West ICB
- NHS Cambridgeshire And Peterborough ICB
- NHS Cheshire And Merseyside ICB
- NHS Cornwall And The Isles Of Scilly ICB
- NHS Coventry And Warwickshire ICB NHS Derby And Derbyshire ICB
- NHS Devon ICB
- NHS Dorset ICB
- NHS Frimley ICB
- NHS Gloucestershire ICB
- NHS Greater Manchester ICB
- NHS Hampshire And Isle Of Wight ICB
- NHS Herefordshire And Worcestershire ICB
- NHS Hertfordshire And West Essex ICB
- NHS Humber And North Yorkshire ICB
- NHS Kent And Medway ICB
- NHS Lancashire And South Cumbria ICB
- NHS Leicester, Leicestershire And Rutland ICB NHS Lincolnshire ICB
- NHS Mid And South Essex ICB
- NHS Norfolk And Waveney ICB
- NHS North Central London ICB
- NHS North East And North Cumbria ICB
- NHS North East London ICB
- NHS North West London ICB
- NHS Northamptonshire ICB
- NHS Nottingham And Nottinghamshire ICB
- NHS Shropshire, Telford And Wrekin ICB NHS Somerset ICB
- NHS South East London ICB
- NHS South West London ICB
- NHS South Yorkshire ICB
- NHS Staffordshire And Stoke-On-Trent ICB
- NHS Suffolk And North East Essex ICB
- NHS Surrey Heartlands ICB
- NHS Sussex ICB
- NHS West Yorkshire ICB

1. If you work for an ICB, does your organisation have a medicines management/medicines optimisation team or a Commissioning Support Unit? *(Tick one)*

- A medicines management/medicines optimisation team only
- A Commissioning Support Unit only
- Both a medicines management/medicines optimisation team and Commissioning Support Unit

1. If you work within a PCN or GP practice, please name the PCN that is your current employer:

|  |
| --- |

1. If you work within a PCN, which ICB oversees your PCN? *(Required, tick one)*

- NHS Bath And North East Somerset, Swindon And Wiltshire ICB
- NHS Bedfordshire, Luton And Milton Keynes ICB
- NHS Birmingham And Solihull ICB
- NHS Black Country ICB
- NHS Bristol, North Somerset And South Gloucestershire ICB
- NHS Buckinghamshire, Oxfordshire And Berkshire West ICB
- NHS Cambridgeshire And Peterborough ICB
- NHS Cheshire And Merseyside ICB
- NHS Cornwall And The Isles Of Scilly ICB
- NHS Coventry And Warwickshire ICB NHS Derby And Derbyshire ICB
- NHS Devon ICB
- NHS Dorset ICB
- NHS Frimley ICB
- NHS Gloucestershire ICB
- NHS Greater Manchester ICB
- NHS Hampshire And Isle Of Wight ICB
- NHS Herefordshire And Worcestershire ICB
- NHS Hertfordshire And West Essex ICB
- NHS Humber And North Yorkshire ICB
- NHS Kent And Medway ICB
- NHS Lancashire And South Cumbria ICB
- NHS Leicester, Leicestershire And Rutland ICB NHS Lincolnshire ICB
- NHS Mid And South Essex ICB
- NHS Norfolk And Waveney ICB
- NHS North Central London ICB
- NHS North East And North Cumbria ICB
- NHS North East London ICB
- NHS North West London ICB
- NHS Northamptonshire ICB
- NHS Nottingham And Nottinghamshire ICB
- NHS Shropshire, Telford And Wrekin ICB NHS Somerset ICB
- NHS South East London ICB
- NHS South West London ICB
- NHS South Yorkshire ICB
- NHS Staffordshire And Stoke-On-Trent ICB
- NHS Suffolk And North East Essex ICB
- NHS Surrey Heartlands ICB
- NHS Sussex ICB

1. If you work in another type of organisation, who is your employer?

|  |
| --- |

1. Which of these best describes your role? *(Tick one)*

- Antimicrobial stewardship lead
- Antimicrobial stewardship pharmacist
- Clinical pharmacist
- Community pharmacist
- GP
- Head of medicines management
- Medicines optimisation pharmacist
- Practice manager
- Practice nurse
- Other

If you selected other, please specify:

|  |
| --- |

1. Are you responsible for antimicrobial stewardship activities in your organisation? *(Tick one)*
   - Yes
   - No

If no, are you aware of an individual responsible for antimicrobial stewardship activities in your organisation? If so, please provide contact details here:

|  |
| --- |

1. Would you consider yourself an AMR ‘champion’, or have you pledged to be an Antibiotic Guardian in the Antibiotic Guardian Campaign? *(Tick one)*
   - Yes
   - No
2. Please briefly describe your roles, responsibilities and priorities in relation to antimicrobial stewardship.

|  |
| --- |

*The following questions are asking about your team's activities over the past 2 years (October 2021 to October 2023).*

Workforce and governance interventions

**Definition**: Changes to organisational structures and job roles that are involved in antibiotic prescribing in some way, such as clinical pharmacists in primary care

1. Within your organisation, please indicate for each of the staff groups below, approximately how many hours of staff time per month are protected to work exclusively on AMS activities (such as education and training, prescribing data analysis and feedback, auditing, quality improvement projects, implementing AMS interventions).

|  | None (zero hours protected time) | 28 hours or less (approx. 1 person for 1 day per week) | Between 28-84 hours (approx. 1 person for 1 to 3 days per week) | 140 hours or more (approx. 1 person working full time) | No such role in my organisation | I don’t know |
| --- | --- | --- | --- | --- | --- | --- |
| Head of medicines management / optimisation |  |  |  |  |  |  |
| Medicines management / optimisation pharmacist |  |  |  |  |  |  |
| PCN pharmacist |  |  |  |  |  |  |
| Medicines management / optimisation pharmacy technicians |  |  |  |  |  |  |
| Other pharmacist roles |  |  |  |  |  |  |
| Clinical director |  |  |  |  |  |  |
| GPs |  |  |  |  |  |  |
| Nurses |  |  |  |  |  |  |
| Data and digital staff |  |  |  |  |  |  |
| Admin staff |  |  |  |  |  |  |

1. Are there any other staff members involved in AMS not listed above? Please list up to 3 and approximately how many hours of staff time per month they have protected to work exclusively on AMS activities.

|  |
| --- |

1. What governance structures exist in your organisation that provide oversight of the quality of antimicrobial prescribing? *(Tick all that apply)*

- A representative from my organisation attends an ICS-wide AMS committee
- Individual prescribers are challenged
- A representative from my organisation is involved in the regional AMS group

1. How would you report concerns around AMS in your organisation?

|  |
| --- |

Diagnostics and prescriber tools

**Definition:** Devices, strategies and tests used by clinicians at the moment of prescription which can affect decisions, such as diagnostic tests.

1. How often do you, or your team, use the following diagnostics during the prescribing process?

|  | Daily | Weekly | Monthly | Every few months, or less | I’m aware of it but we don’t use it | I haven’t heard of it |
| --- | --- | --- | --- | --- | --- | --- |
| C-Reactive Protein (CRP) |  |  |  |  |  |  |
| Dip stick tests for UTIs |  |  |  |  |  |  |
| Procalcitonin (PCT) |  |  |  |  |  |  |

1. Are there any other diagnostics you use that are not listed above? Please list up to 3 and approximately how often you use them.

|  |
| --- |

1. Which diagnostics do you think are most useful to help optimal prescribing? *(Tick one)*
   - C-Reactive Protein (CRP)
   - Dip stick tests for UTIs
   - Procalcitonin (PCT)

|  |
| --- |

1. How often do you, or your organisation, use the following tools and prompts during the prescribing process?

|  | Daily | Weekly | Monthly | Every few months, or less | I’m aware of it but we don’t use it | I haven’t heard of it |
| --- | --- | --- | --- | --- | --- | --- |
| Clinical prediction scores (e.g. FeverPAIN, Centor, STARWAVe) |  |  |  |  |  |  |
| Computerised decision support tools |  |  |  |  |  |  |
| Delayed (back-up) prescribing |  |  |  |  |  |  |
| Digital prompts and reminders |  |  |  |  |  |  |
| Digital safety alerts |  |  |  |  |  |  |
| Text messages to patients |  |  |  |  |  |  |

1. Are there any other tools you use that are not listed above? Please list up to 3 and approximately how often you use them.

|  |
| --- |

1. Which tools do you think are most useful to help optimal prescribing? *(Tick one)*
   - Clinical prediction scores (e.g. FeverPAIN, Centor, STARWAVe)
   - Computerised decision support tools
   - Delayed (back-up) prescribing
   - Digital prompts and reminders
   - Digital safety alerts
   - Text messages to patients

Public awareness interventions

**Definition:** Strategies to engage and educate people who are not experts or professionals in AMR, such as TV adverts, posters and leaflets

1. Which of the following interventions have you or your team used to make the public or patients more aware of appropriate antibiotic use?

|  | Weekly or more | Monthly | Every few months, or less | I’m aware of it but we don’t use it | I haven’t heard of it |
| --- | --- | --- | --- | --- | --- |
| Conversations directly with patients |  |  |  |  |  |
| Posters or infographics |  |  |  |  |  |
| Leaflets or other printed information (e.g. TARGET self-care leaflet) |  |  |  |  |  |
| Activities around World Antibiotic Awareness Week |  |  |  |  |  |
| Animated films |  |  |  |  |  |
| Signposting patients to internet resources |  |  |  |  |  |
| Text messages to patients |  |  |  |  |  |

1. Are there any other patient awareness strategies which you use that are not listed above? Please list up to 3 and approximately how often you use them.

|  |
| --- |

Professional engagement and training

**Definition:** Educational and awareness-raising interventions that target professionals (e.g. doctors, nurses, pharmacists, medical students), such as conferences and courses. Example: TARGET training, e-learning

1. How many staff within your organisation* have taken part in AMS-related professional engagement activities over the last 2 years, including training, conferences?

I*f you work at an ICB level, please say how many staff within your medicines management or medicines optimisation team. If you work at a PCN level, please say how many staff within the PCN*

|  | 0 | 1 | 2 | 3 or more | I don’t know |
| --- | --- | --- | --- | --- | --- |
| Short events and webinars lasting under 2 hours |  |  |  |  |  |
| Half-day events |  |  |  |  |  |
| Whole-day events |  |  |  |  |  |
| Multi-day events |  |  |  |  |  |
| Formal courses leading to accreditation (e.g. FutureLearn courses) |  |  |  |  |  |

1. Are there any other training opportunities that staff in your organisation have taken part in? Please list up to 3 and approximately how often you use them.

|  |
| --- |

1. Which of these have been most useful? *(Tick one)*
   - Short events and webinars lasting under 2hours
   - Half-day events
   - Whole-day events
   - Multi-day events
   - Formal courses leading to accreditation (e.g. FutureLearn courses)
2. Please provide an example of a specific event or opportunity if possible.

|  |
| --- |

Guidance and toolkit interventions

**Definition of guidance and toolkits:** Resources used by healthcare professionals and antimicrobial stewardship teams to support delivery of appropriate care and services, such as guidelines for specific indications. Examples: TARGET toolkit, NICE guidelines

|  | Weekly or more | Monthly | Every few months | Annually or less | I’m aware of it but we don’t use it | I haven’t heard of it |
| --- | --- | --- | --- | --- | --- | --- |
| Guidelines produced by Royal Colleges (e.g. Royal College of General Practitioners, Royal College of Nursing, Royal Pharmaceutical Society) |  |  |  |  |  |  |
| Local antibiotic guidelines |  |  |  |  |  |  |
| NHSE AMR programme FutureNHS workspace |  |  |  |  |  |  |
| NICE infection management and antimicrobial prescribing guidance |  |  |  |  |  |  |
| NICE stewardship guidance (NG15) [1] |  |  |  |  |  |  |
| TARGET toolkit resources [2] |  |  |  |  |  |  |
| UKHSA summary of antimicrobial prescribing guidance table (managing common infections) [4] |  |  |  |  |  |  |

1. Over the last 2 years, how often have you used each of the following resources?

*[1] NICE guidance: https://www.nice.org.uk/guidance/ng15
[2] TARGET resources: https://elearning.rcgp.org.uk/course/view.php?id=553
[3] TARGET How to..? https://elearning.rcgp.org.uk/mod/book/view.php?id=12649&chapterid=793
[4] UKHSA antimicrobial prescribing guidance: https://elearning.rcgp.org.uk/mod/book/view.php?id=12648&chapterid=453*

1. Are there any other examples of guidance documents or toolkits for AMS not mentioned above? Please list up to 3 and approximately how often you use them.

|  |
| --- |

1. Out of the resources selected above, which is the most useful for you? *(Tick one)*
   - Guidelines produced by Royal Colleges (e.g. Royal College of General Practitioners, Royal College of Nursing, Royal Pharmaceutical Society)
   - Local antibiotic guidelines
   - NHSE AMR programme FutureNHS workspace
   - NICE infection management and antimicrobial prescribing guidance
   - NICE stewardship guidance (NG15)
   - TARGET toolkit resources
   - TARGET How to...? guides for recurrent infection
   - UKHSA summary of antimicrobial prescribing guidance table (managing common infections)

Monitoring and feedback interventions

**Definition:** Interventions based on the collection, analysis, interpretation and communication of data to inform prescribers about patterns in antimicrobial consumption, using data platforms and dashboards. Examples: ePACT2, PrescQIPP, Fingertips, audits

1. Over the last 2 years, how often have you or your team used the following data sources, for example during meetings, to identify ways to improve or inform decisions about prescribing?

|  | Weekly or more | Monthly | Every few months | Annually or less | I’m aware of it but we don’t use it | I haven’t heard of it |
| --- | --- | --- | --- | --- | --- | --- |
| Data from GP clinical systems e.g. EMIS Web, SystmOne Vision |  |  |  |  |  |  |
| ePACT2 [1] |  |  |  |  |  |  |
| Fingertips [2] |  |  |  |  |  |  |
| Model Health System [3] |  |  |  |  |  |  |
| NHS Oversight Framework metrics (FutureNHS) [4] |  |  |  |  |  |  |
| OpenPrescribing [5] |  |  |  |  |  |  |
| PharmOutcomes [6] |  |  |  |  |  |  |
| PrescQIPP [7] |  |  |  |  |  |  |
| Primary Care Indicators Dashboard - General Practice Indicators [8] |  |  |  |  |  |  |

*[1] ePACT2* [*https://www.nhsbsa.nhs.uk/access-our-data-products/epact2*](https://www.nhsbsa.nhs.uk/access-our-data-products/epact2) *[2] Fingertips* [*https://fingertips.phe.org.uk*](https://fingertips.phe.org.uk) *[3] Model Health System* [*https://model.nhs.uk*](https://model.nhs.uk) *[4] NHS Oversight Framework metrics (FutureNHS)* [*https://future.nhs.uk/A_M_R/view?objectId=33692656*](https://future.nhs.uk/A_M_R/view?objectId=33692656)

*[5] OpenPrescribing* [*https://openprescribing.net*](https://openprescribing.net)

*[6] PhamOutcomes* [*https://pharmoutcomes.org/pharmoutcomes/*](https://pharmoutcomes.org/pharmoutcomes/) *[7] PrescQIPP* [*https://www.prescqipp.info*](https://www.prescqipp.info) *[8] Primary Care Indicators Dashboard* [*https://www.primarycareindicators.nhs.uk/*](https://www.primarycareindicators.nhs.uk/)

1. Are there any other data sources you use that are not listed above? Please list up to 3 and approximately how often you use them.

|  |
| --- |

1. How often do you or your team conduct audits of antibiotic prescribing?

|  | Monthly, or more | Every few months | Annually or less | I’m aware of it but we don’t use it | I haven’t heard of it |
| --- | --- | --- | --- | --- | --- |
| Audits initiated by ICBs |  |  |  |  |  |
| Audits initiated by GP or PCN staff |  |  |  |  |  |
| Audits from the TARGET toolkit |  |  |  |  |  |
| Audits on broad spectrum prescribing |  |  |  |  |  |

1. Are there any other types of audits conducted that are not listed above? Please list up to 3 and approximately how often these are conducted.

|  |
| --- |

1. Which of these data sources or audits are most useful for you? *(Tick one)*
   - Data from GP clinical systems e.g. EMIS Web, SystmOne Vision
   - ePACT2
   - Fingertips
   - Model Health System
   - NHS Oversight Framework metrics (FutureNHS)
   - OpenPrescribing
   - PharmOutcomes
   - PrescQIPP
   - Primary Care Indicators Dashboard - General Practice Indicators
   - Audits initiated by ICBs
   - Audits initiated by GP or PCN staff
   - Audits from the TARGET toolkit
   - Audits on broad spectrum prescribing
2. Please explain why that data source is most useful to you:

|  |
| --- |

Policy and commissioning interventions

**Definition**: Interventions involving the planning, prioritising, and purchasing of services to achieve specific goals, such as quality improvement, efficiency, or financial incentive mechanisms. Example: Quality Premium, NHS Oversight Framework

1. How relevant are the following interventions that aim to incentivise optimal antimicrobial prescribing to your work?

|  | Very relevant. It affects our work weekly or more | Somewhat relevant. It affects our work every few months | I’m aware of it but it is not relevant to me | I haven’t heard of it |
| --- | --- | --- | --- | --- |
| NHS Oversight Framework (NOF) [1] |  |  |  |  |
| ICB- or sub-ICB-led local incentive schemes |  |  |  |  |
| PCN Network contract Directed Enhanced Service (DES) [2] |  |  |  |  |
| GP contract [3] |  |  |  |  |
| Medicines Optimisation Opportunities [4] |  |  |  |  |

*[1] NHS Oversight Framework* [*https://www.england.nhs.uk/publication/nhs-oversight-framework-22-23/*](https://www.england.nhs.uk/publication/nhs-oversight-framework-22-23/) *[2] PCN Network contract Directed Enhanced Service (DES)* [*https://www.england.nhs.uk/gp/investment/gp-contract/network-contract-directed- enhanced-service-des/*](https://www.england.nhs.uk/gp/investment/gp-contract/network-contract-directed-%20enhanced-service-des/) *[3] GP contract* [*https://www.england.nhs.uk/gp/investment/gp-contract/*](https://www.england.nhs.uk/gp/investment/gp-contract/) *[4] Medicines Optimisation Opportunities* [*https://www.england.nhs.uk/long-read/national-medicines-optimisation-opportunities-2023-24/*](https://www.england.nhs.uk/long-read/national-medicines-optimisation-opportunities-2023-24/)

1. Are there any other incentives that you are aware of which are not listed above? Please list up to 3 and approximately how often they affect your work.

|  |
| --- |

1. How often does the UK’s 5-year AMR National Action Plan (NAP) affect your work? *(Tick one)*

- The NAP affects our work, on a weekly basis or more
- The NAP affects our work every few months
- I’m aware of the NAP but it is not relevant to me
- I haven’t heard of it

Classification systems

**Definition:** Interventions based on the categorisation of elements relevant to the antibiotic use system, such as drug classification systems

1. Do you use, or have you heard of, the World Health Organization's AWaRe classification system*? For example, in monitoring and reporting antibiotic use by AWaRe categories, or as part of prescribing guidance. *(Tick one)*

**AWaRe categorises antibiotics into three groups: Access (first- and second-choice antibiotics for treating common infections), Watch (antibiotics with a higher resistance potential) and Reserve (last resort treatments). The UK was the first country to include the AWaRe categorisation in its National Action Plan and for informing AMS programmes https://aware.essentialmeds.org/list*

- Yes, it is part of our regular work (on a weekly basis or more)
- Yes, it is part of our work occasionally
- I’ve heard of it, but we don’t use it
- No, I haven’t heard of it
- I don’t know

1. If yes, what situations does AWaRe come into your work?

|  |
| --- |

Summary

1. Please rank the following types of intervention by order of what you think has the most influence on antimicrobial prescribing, from 1 to 7 (1 being most useful, 7 being least impactful):

|  | 1 (most impactful) | 2 | 3 | 4 | 5 | 6 | 7 (least impactful) |
| --- | --- | --- | --- | --- | --- | --- | --- |
| Guidance and toolkits (e.g. TARGET) |  |  |  |  |  |  |  |
| Monitoring and feedback (e.g. data, audits) |  |  |  |  |  |  |  |
| Policy & commissioning (e.g. incentive mechanisms like the quality premium) |  |  |  |  |  |  |  |
| Prescriber tools (e.g. diagnostic tests, delayed prescribing) |  |  |  |  |  |  |  |
| Professional engagement and training (e.g. courses) |  |  |  |  |  |  |  |
| Workforce (new AMS- specific roles) |  |  |  |  |  |  |  |
| Public awareness (information for the public, campaigns) |  |  |  |  |  |  |  |

Final page

Thank you for taking the time to complete the survey.

We will be in touch if you have opted in to hearing the findings from the survey.

## B – Interventions used in primary care in England

### **Table S1: Description of interventions asked about in the survey**

**indicates the interventions which were identified as part of a combination that occurred in ICBs that met prescribing targets*

| **Category** | | **Intervention** | **Description and link if available** |
| --- | --- | --- | --- |
| 1. **Guidelines and toolkits** | | Guidelines produced by Royal Colleges | Guidance from professional bodies including the Royal College of General Practitioners, Royal College of Nursing, Royal Pharmaceutical Society RCGP, RCN, and RPS to support best practice in prescribing. |
|  |  | *Local antibiotic guidelines | Locally adapted prescribing guidance tailored to regional resistance patterns and formulary. (Usually available via local ICB or NHS Trust websites) |
|  |  | NHSE AMR programme FutureNHS workspace | An online collaboration platform that serves as a central hub for sharing AMR resources, information, guidance, data, and dashboards. It is available for NHS staff and other professionals invited to collaborate.  <https://future.nhs.uk/A_M_R/view?objectId=33692656> |
|  |  | *NICE infection management and antimicrobial prescribing guidance | The National Institute for Health and Care Excellence (NICE) provides evidence-based national guidance and advice. This guidance is a suite of evidence-based guidelines for managing common infection syndromes in the context of AMR. <https://www.nice.org.uk/Media/Default/About/what-we-do/NICE-guidance/antimicrobial%20guidance/scope-antimicrobial-prescribing-guidelines.pdf> |
|  |  | NICE stewardship guidance (NG15) | NICE guidance that provides general advice and guidance on prescribing and stewardship to slow the emergence of AMR. It does not cover specific clinical conditions or name medicines. <https://www.nice.org.uk/guidance/ng15> |
|  |  | TARGET toolkit resources | TARGET stands for Treat Antibiotics Responsibly, Guidance, Education and Tools. It is a toolkit designed to support primary care clinicians to champion and implement antimicrobial stewardship activities. It includes guidance on how to discuss antibiotics with patients, specific advice on some infection syndromes (e.g. urinary tract infections, respiratory tract infections), and learning resources for prescribers. <https://elearning.rcgp.org.uk/course/view.php?id=553> |
|  |  | *UKHSA summary of antimicrobial prescribing guidance table (managing common infections) | UKHSA (formerly Public Health England) worked with NICE to produce a table of managing common infections. This contained descriptions of symptoms, diagnosis, evidence and treatment recommendations for common syndromes.  <https://www.gov.uk/government/publications/managing-common-infections-guidance-for-primary-care> |
| 1. **Monitoring and feedback** | **Data** | Data from GP clinical systems e.g. EMIS Web, SystmOne Vision | Electronic Patient Record (EPR) systems used by GP practices in England, which includes patient and prescribing data. Several EPR systems exist, the most used systems being EMIS Helah and SystmOne. |
|  |  | *ePACT2 | An online data tool provided by the NHS Business Services Authority and accessible for the NHS authorised users with access to data to analyse prescribing trends, improve patient care, and support medicines management. It has numerous dashboards and reports, and can provide information on high-level summaries and individual prescription details. <https://www.nhsbsa.nhs.uk/access-our-data-products/epact2> |
|  |  | *Fingertips | Fingertips provides openly available, summarised data for public health professionals. The AMR indicators are produced by UKHSA and cover trends in antibiotic prescribing, AMR, infection prevention control and stewardship. Data is mostly presented at the ICB or regional level. <https://fingertips.phe.org.uk> |
|  |  | Model Health System | A data-driven improvement tool that enables NHS health systems and trusts to benchmark quality and productivity. It is accessible to all NHS staff. <https://model.nhs.uk> |
|  |  | NHS Oversight Framework metrics (FutureNHS) | The system of indicators and metrics NHS England uses to assess performance of NHS Trusts and Integrated Care Boards (ICBs). For ICB-level antibiotic prescribing, there are two metrics:   - Total antibiotic prescribing in primary care (items per STAR-PU) — target ≤ 0.871 items per STAR-PU. - Proportion of broad-spectrum antibiotics (co-amoxiclav, cephalosporins, quinolones) in total antibiotic prescribing — target ≤ 10%.   The data on these metrics is reported via a dashboard on the FutureNHS collaboration platform (access available for NHS users and at request by others): <https://future.nhs.uk/A_M_R/view?objectId=33692656> |
|  |  | OpenPrescribing | Publicly available website run by the Bennett Institute for Applied Data Science at the University of Oxford presenting data on prescribing. The data is from the NHS Business Services Authority and is presented at either the GP practice, Primary Care Network, ICB or regional level in a visual, searchable and transparent way. <https://openprescribing.net> |
|  |  | PharmOutcomes | A web-based platform for pharmacy services and data reporting, mostly used by community pharmacies and NHS commissioners to record clinical services, track outcomes, and generate claims for payment. <https://pharmoutcomes.org/pharmoutcomes/> |
|  |  | PrescQIPP | An online data platform provided by a not-for-profit company to support the NHS. It produces prescribing dashboards, data packs, audit templates, guidance and tools to help clinicals and medicines optimisation teams improve prescribing. <https://www.prescqipp.info> |
|  |  | Primary Care Indicators Dashboard - General Practice Indicators | An NHS tool to monitor and benchmark general practice performance, primarily for ICBs to help them manage, commission and improve GP services. It is also used by NHS England for oversight, assurance and to track variation in GP performance. It includes medicines management (prescribing), workforce (staff, capacity), and clinical outcomes data. Access is available for NHS users. <https://www.primarycareindicators.nhs.uk/> |
|  | **Audits** | *ICB-led audits | Structured reviews of prescribing patterns, run by the Integrated Care Board (ICB) |
|  |  | GP or PCN-led audits | Structured reviews of prescribing patterns, run by GP practices or Primary Care Networks (PCNs). |
|  |  | Audits from the TARGET toolkit | Structured reviews of prescribing patterns, designed by UKHSA and available through the TARGET toolkit online for GP practices, PCNs and community pharmacists to use. <https://elearning.rcgp.org.uk/mod/book/view.php?id=12649&chapterid=454> |
|  |  | Broad spectrum prescribing audits | Structured reviews of prescribing patterns focusing on how often and how appropriately clinicians prescribe broad-spectrum antibiotics like co-amoxiclav, cephalosporins and quinolones. |
| 1. **Policy, commissioning and incentives** | | National Action Plan (NAP) for AMR | A UK-wide strategy to address the threat of drug-resistant infections. The current plan for 2024-2029 is the second in a series designed to deliver the UK’s 20-year vision for AMR to be contained and controlled by 2040.  <https://www.gov.uk/government/publications/uk-5-year-action-plan-for-antimicrobial-resistance-2024-to-2029/confronting-antimicrobial-resistance-2024-to-2029> |
|  |  | *NHS Oversight Framework (NOF) | An approach used for assessing ICBs and hospital trusts to monitor performance and improve public accountability. ICBs are generally scored and ranked into ‘segments’ reflecting their performance, based on the aggregation of data from metrics for a number of areas. By assessing ICBs across the country in this way, it acts as an incentive.  <https://www.england.nhs.uk/publication/nhs-oversight-framework-22-23/> |
|  |  | *ICB- or sub-ICB-led local incentive schemes | Mechanisms used, financial or otherwise, by ICBs to encourage clinicians within their area to improve how much and how appropriately they prescribe antibiotics. |
|  |  | Primary Care Networks: network contract Directed Enhanced Service (DES) | The DES brings GP practices together to work at a larger scale under Primary Care Networks (covering populations of approximately 30,000 to 50,000 patients), and funds services beyond the core GP contract to meet specific needs of a local community. It promotes and funds enhanced AMS programmes and affects antibiotic use through:   - Additional Roles Reimbursement Scheme (ARRS) allowing PCNs to employ clinical pharmacists, pharmacy technicians (amongst others) who play a pivotal role in AMS by leading prescribing audits, guideline implementation and reviewing antibiotic prescribing practices - Structured Medication Reviews which require reviewing patients with complex needs, which often includes a review of long-term or inappropriate antibiotic regimens - Data-driven approaches to enable population health management, including using prescribing data to identify high-volume or high-risk antibiotic prescribers and target interventions effectively (using tools like ePACT2 and OpenPrescribing) - Encouraging PCNs to collaborate with community pharmacists (and other service providers in the local area)   [https://www.england.nhs.uk/gp/investment/gp-contract/network-contract-directed- enhanced-service-des/](https://www.england.nhs.uk/gp/investment/gp-contract/network-contract-directed-%20enhanced-service-des/) |
|  |  | GP contract | An agreement between the NHS and GP practices to provide primary medical services to the public. It defines the core services practices must offer, the standards they must meet and how they are funded. For antibiotic use, it:   - Defines the core services for infection management - Shapes workforce and access requirements e.g. length of consultations, who can prescribe and reducing waiting times - Has incentive schemes and performance frameworks that influence prescribing paper (such as the Quality and Outcomes Framework (which rewards practices for achieving specific targets, including historically indicators related to prescribing)   <https://www.england.nhs.uk/gp/investment/gp-contract/> |
|  |  | *Medicines Optimisation Opportunities | A national approach to support ICBs and systems improve patient outcomes by asking ICBs to identify at least 5 opportunities to focus on to improve medicine use, depending on their local system opportunities and population needs. The national guidance provides a list of 16 possible opportunities ICBs can pick from, including the stewardship ambitions of reducing course length of antimicrobial prescribing and switching intravenous antibiotic to oral.  <https://www.england.nhs.uk/long-read/national-medicines-optimisation-opportunities-2023-24/> |
|  |  | AWaRE classification | The AWaRE classification is a tool for policy makers, researchers, and healthcare professionals to monitor antibiotic consumption, defining targets and monitoring the effects of stewardship policies. Set by WHO, the UK has an adapted version relevant to prescribing in the UK. The antibiotics are grouped as:   - Access: antibiotics with a narrow spectrum of activity, fewer side effects, lower costs and lower resistance potential - Watch: antibiotics with higher resistance potential and are broader spectrum, which should be the first or second choice antibiotics for a limited number of infective syndromes - Reserve: last resort antibiotics used for highly selected patients   <https://www.gov.uk/government/publications/uk-aware-antibiotic-classification/uk-access-watch-reserve-and-other-classification-for-antibiotics-uk-aware-antibiotic-classification> |
| 1. **Workforce and governance** | **Time protected for staff to spend on AMS** | Head of medicines management / optimisation | Time protected for a senior pharmacist to spend on AMS activities. This role provides expert clinical and strategic leadership to ensure the safe, effective, cost-efficient use of medicines across an ICB. |
|  |  | Medicines management / optimisation pharmacist | Time protected for a clinical pharmacist to spend on AMS activities. These clinical pharmacists work on improving the way medicines are used to achieve the best outcomes for patients, working in primary care, ICBs and NHS trusts. They work directly with GP teams and patients to review medications, support appropriate prescribing, run clinics independently or alongside GPs, and handle prescribing safety governance (e.g. audits). |
|  |  | PCN pharmacist | Time protected for PCN pharmacists to spend on AMS activities. These roles are medicines optimisation pharmacists specifically based in Primary Care Networks to provide expert medicines support across all GP practices in their network. |
|  |  | Medicines management / optimisation pharmacy technicians | Time protected for trained pharmacy professional to spend on AMS activities. They are not prescribers but instead are highly skilled in medicines systems and support clinical pharmacists, for example by assessing repeat prescriptions, running audits, creating reports, reducing medicines waste, and providing safety checks. |
|  |  | Other pharmacists | Time protected for any other pharmacist role to spend on AMS activities. |
|  |  | Clinical director | Time protected for one of the senior clinical leadership roles in the management of an ICB, to deliver AMS activities. They act as a bridge between clinical practice and system-wide commissioning, providing clinical leadership and strategy |
|  |  | GPs | Time protected for a General Practitioner (medical doctor who works in primary care) to spend on AMS activities. |
|  |  | Nurses | Time protected for a nurse to spend on AMS activities. |
|  |  | Data and digital staff | Time protected for staff involved in data analysis or digital technology to spend on AMS activities, including analysing prescribing patterns, resource allocation, service performance, quality, and safety. |
|  |  | Admin staff | Time protected for administrative staff to work on AMS activities in ICBs. |
|  | **Governance** | Representative attending an ICS-wide AMS committee | With Integrated Care Systems (ICS), there is an multidisciplinary group of healthcare professionals and stakeholders responsible for coordinating and implementing a strategic approach to antimicrobial stewardship across all care settings within a specific geographical area, covering both primary and secondary care. |
|  |  | *Challenging individual prescribers | Using information from data, audits and guidelines to question prescribers on particular patterns or habits of prescribing, in order to try to reduce unnecessary prescribing. Pharmacists and other clinicians are encouraged to establish a dialogue with prescribers to discuss concerns directly and build trust. |
|  |  | Representative involved in regional AMS group | There are seven Regional AMS Leads in NHS England who each run an AMS group for their geographic region, and which ICB representatives are encouraged to attend. |
| 1. **Prescriber tools and diagnostics** | **Diagnostics** | C-Reactive Protein (CRP) | A blood test that measures the level of C-reactive protein, a non-specific marker released into the blood in response to infections and inflammatory triggers. It is an indicator of inflammation and used to guide antibiotic prescribing for lower respiratory tract infections as it can help differentiate viral and self-limiting infections from more serious bacterial infections that need antibiotics.  <https://www.nice.org.uk/advice/mib78/chapter/the-technology> |
|  |  | Dip stick tests for UTIs | Point of care tests that are made of small plastic strips with chemical pads which change colour when dipped in a urine sample, used to help diagnose urinary tract infections for certain groups of the population.  <https://www.gov.uk/government/publications/urinary-tract-infection-diagnosis/diagnosis-of-urinary-tract-infections-quick-reference-tools-for-primary-care> |
|  |  | Procalcitonin (PCT) | A blood test measuring level of procalcitonin, a biomarker indicating the presence of bacterial and viral infections, for example for helping manage patients with suspected sepsis or pneumonia. |
|  | **Tools used at the moment of prescription** | Clinical prediction scores (e.g. FeverPAIN, Centor, STARWAVe) | A simple tool designed to help guide healthcare professionals make decisions around diagnosis, prognosis and treatment decisions, based on combining multiple patient characteristics and clinical observations into a single objective score:   - FeverPAIN is a score used in the UK for sore throat. There are five criteria: (1) ever during previous 24 hours, (2) purulence or pus on tonsils, (3) attend rapidly within 3 days after onset of symptoms, (3) severely inflamed tonsils, (5) no cough. Each criteria scores 1 point and the higher scores suggest more severe symptoms and likely bacterial (streptococcal) cause. - Centor scores are also used to assess sore throat, with four criteria: (1) tonsillar exudate, (2) tender anterior cervical lymphadenopathy or lymphadenitis, (3) history of fever, and (4) absence of cough. Higher scores indicate likelihood of isolating streptococcus. - STARWAVe has seven clinical criteria, used to assess children with an acute cough or respiratory tract infection. The criteria are (1) short illness duration, less than 3 days, (2) temperature, documented fever (3) age, children under 2 years, (4) recession, (5) wheeze, (6) asthma history, (7) vomiting.   <https://www.nice.org.uk/guidance/ng84/chapter/terms-used-in-the-guideline>  <https://www.nice.org.uk/guidance/ng250/documents/evidence-review-12> |
|  |  | *Computerised decision support tools | A software application designed to assist healthcare professionals in making evidence-based decisions about patient care. These tools combine patient data with clinical knowledge and algorithms to generate patient=specific recommendations.  <https://www.england.nhs.uk/long-read/supporting-clinical-decisions-with-health-information-technology/> |
|  |  | *Delayed (back-up) prescribing | a strategy used by healthcare professionals to manage conditions that are likely to get better on their own. Instead of providing an antibiotic immediately, a prescriber gives the patient a prescription with instructions to only have it dispensed and used if their symptoms worsen or have not improved after a specific number of days. <https://www.nice.org.uk/guidance/qs121/chapter/quality-statement-2-back-up-delayed-prescribing> |
|  |  | Digital prompts and reminders | Digital tools integrated into clinical and prescribing systems that enable clinicians to follow prescribing guidance or support around appropriate treatment choices and durations.  <https://www.england.nhs.uk/long-read/digital-vision-for-antimicrobial-stewardship-in-england/> |
|  |  | Digital safety alerts | Digital alerts that are integrated into electronic health record and prescribing systems to provide clinicals with patient-specific information to prevent medication errors, minimise harm and standardise best practice. |
|  |  | Text messages to patients | GPs can send targeted texts to individual patients to help them manage them manage their prescription and offer post-consultation support, for example if they have been given a delayed prescription, a text message can be sent to reinforce advice given by the GP. |
| 1. **Public awareness** | | Conversations directly with patients | Clinicians and pharmacists are encouraged to discuss the benefits and risks of antibiotic prescribing with their patient to help them understand the importance of rational use of antibiotics and particularly that antibiotics will not work for common coughs and colds caused by viruses. |
|  |  | Posters or infographics | Posters put up in GP practices or community pharmacies with messaging to help increasing awareness of the importance of appropriate antibiotic use. Sources of posters include the Keep Antibiotics Working campaign, Antibiotic Guardian campaign, the TARGET toolkit resources, and others produced for specific awareness-raising events:  <https://antibioticguardian.com/resources/posters-and-leaflets/>  <https://campaignresources.dhsc.gov.uk/campaigns/keep-antibiotics-working/> |
|  |  | Leaflets or other printed information (e.g. TARGET self-care leaflet) | Leaflets to help prescribers discuss antibiotics and their symptoms in consultations, including those produced by the Royal College of GPs and UKHSA in the TARGET toolkit on self-care, respiratory tract infections, and urinary tract infections: <https://elearning.rcgp.org.uk/mod/book/view.php?id=12647&chapterid=440> |
|  |  | Activities around World Antimicrobial Awareness Week | Every year in November, there is a World Antimicrobial Awareness Week (WAAW) and European Antimicrobial Awareness Day, coordinated by the World Health Organization to increase awareness of AMR. It promotes best practices among the public and healthcare workers. In England, national and local settings use this opportunity to focus public awareness campaigns,  <https://www.who.int/campaigns/world-amr-awareness-week>  <https://www.england.nhs.uk/long-read/world-antimicrobial-resistance-awareness-week-2024/> |
|  |  | Animated films | Short animations or adverts displayed in healthcare settings to raise awareness about antibiotics, such as the Keep Antibiotics Working campaign. <https://campaignresources.dhsc.gov.uk/campaigns/keep-antibiotics-working/hero-video/> |
|  |  | Signposting patients to internet resources | Either via conversations with patients, posters, leaflets of texts to patients, prescribers can direct patients to resources online, such as the patient information website.  <https://patient.info> |
|  |  | Text messages to patients | Automated, non-specific texts sent to patients with information about antimicrobial stewardship in general, which could include advice on treating common colds during winter months. |
| 1. **Professional engagement and training** | | Short events and webinars lasting under 2 hours | There are numerous webinars available for healthcare staff to train on antimicrobial prescribing, with many resources offered by the NHS, professional bodies like the Royal College of General Practitioners (RCGP), and specialist organisations. The RCGP and UKHSA often run free TARGET antibiotics webinars with recordings available on the RCGP website, the NHS offers a range of online learning resources (e.g. on the e-LfH platform), and the British Society of Antimicrobial Chemotherapy also provides resources.  <https://elearning.rcgp.org.uk/mod/book/view.php?id=14616>  <https://www.e-lfh.org.uk>  <https://bsac.org.uk/events/webinars/> |
|  |  | Half-day events | As above for short events, there can be longer half-day events for healthcare staff to join, either run by their own NHS or ICB organisation or by a national organisation, either run in-person or virtually. |
|  |  | Whole-day events | As above for short events, there can be longer whole-day events for healthcare staff to join, either run by their own NHS or ICB organisation or by a national organisation, either run in-person or virtually. |
|  |  | Multi-day events | There are several multi-day training options for antimicrobial stewardship (AMS) available to healthcare professionals in the UK, often offered by universities and specialist bodies like the British Society for Antimicrobial Chemotherapy (BSAC). Examples include the University of Dundee and BSAC’s Massive Open Online Course (MOOC) on antimicrobial stewardship, the London School of Hygiene and Tropical Medicine 5-day intensive course in AMR. Local training hubs and ICBs can commission or run multi-day training tailored to local needs too.  <https://www.futurelearn.com/courses/antimicrobial-stewardship>  <https://www.lshtm.ac.uk/study/courses/short-courses/antimicrobial-resistance> |
|  |  | Formal courses leading to accreditation (e.g. FutureLearn courses) | Speciality programmes, including Masters degrees, post-graduate courses, as well as courses resulting in other accreditation including and the RCGP TARGET toolkit train the trainer workshop or primary care professionals who wish to become accredited trainers for the TARGET antibiotics toolkit.  <https://elearning.rcgp.org.uk/mod/book/view.php?id=12651> |

## C – fsQCA methods

### **Table S2: Stages of fsQCA**

| QCA step | Purpose | Methods |
| --- | --- | --- |
| - 1. Case selection | Identifying the cases to include in the QCA analysis | All ICBs which responded to the survey were included as cases (29 out of 42 ICBs). |
| - 1. Condition and outcome calibration | To transform the raw numerical data on the conditions and outcome into set membership scores, based thresholds and qualitative/ theoretical knowledge. | Conditions were calibrated indirectly by assigning fuzzy scores between 0 and 1 based on the extent to which an ICB implemented an intervention, using the ordinal responses to survey questions and subject area knowledge^1^. The outcome was antibacterial items prescribed per STAR-PU, calibrated into three levels based on national policy targets (see calibration tables below). |
| - 1. Condition selection | The survey collected data on 61 interventions (conditions). Without some reduction, this would have been an infeasible number of interventions (conditions) to handle in a single QCA model. | Out of a possible 61 interventions that data was collected on in the survey, 49 conditions were selected for initial inclusion:   - Guidance and toolkits: 8 (all) interventions - Monitoring and feedback: 9 interventions (reduced from 13 because only the 5 most used data platforms were included in the fsQCA, along with 4 types of audit. The 5 data systems included were ePACT2, PrescQIPP, OpenPrescribing, Fingertips and data from the GP’s own clinical system) - Professional engagement: 5 (all) interventions - Public awareness: 7 (all) interventions - Policy, incentives and commissioning: 6 (all) interventions - Prescriber tools: 8 (all) interventions - Workforce and governance: 6 interventions (reduced from 13 because firstly the survey asked about 3 types of pharmacists that were then combined and analysed as one 'any pharmacist' intervention. Secondly, 5 other interventions were not included because they had too few responses about staff with time protected for AMS in the following roles: clinical director; data and digital staff; GPs; nurses; or admin staff working on AMS.   These conditions were analysed in category models first (see Step 5 below). |
| - 1. Necessity analysis | It is considered good practice to identify which conditions are necessary to achieve the outcome on their own first because the main QCA steps only analyse sufficiency^2,3^. | The benchmark for testing causal necessity (a condition that is always present when the outcome occurs) was 0.9 for consistency score and 0.5 for coverage score^4^.  One necessary intervention was found: using locally adapted guidance. |
| - 1. Category model construction | To reduce the number of interventions in each category so models which combined categories of interventions could then be developed^3,5^. | The 49 interventions were grouped using categories from a systematic review ^6^. The groups were assessed in models to identify which specific interventions were most influential for achieving the outcome.  Each category model had three to eight specific interventions:   - Guidance and toolkits: 1 model with 8 interventions - Monitoring and feedback: 2 models with 5 data systems in one and 4 types of audits in another - Professional engagement: 1 model with 5 interventions - Public awareness: 1 model with 7 interventions - Policy, incentives and commissioning: 1 model with 6 interventions - Prescriber tools: 2 models with 3 diagnostic tests in one and 5 prescriber tools in another - Workforce and governance: 2 models with 3 staff roles in one and 3 governance structures in another   The threshold for a solution to be considered as causally sufficient (i.e. the combination of conditions was accompanied by the presence of the outcome) was having a consistency score of at least 0.75, as recommended by Rihoux and Ragin^1^.  Solutions from this stage are in Supplemental Table D. This step led to 21 interventions being identified as being sufficient across the categories:   - Guidance and toolkits: 3 interventions - Monitoring and feedback: 5 interventions - Professional engagement: 0 interventions - Public awareness: 0 interventions - Policy, incentives and commissioning: 4 interventions - Prescriber tools: 5 interventions - Workforce and governance: 4 interventions |
| - 1. Pathways hypothesised | To describe how the categories of interventions interact and occur with the outcome, based on existing logic models^7,8^. | All pathways started with either incentives, policy or workforce interventions as these were hypothesised to stimulate and support other interventions. The 7 pathways hypothesised were:  A – incentives + guidance  B – incentives + data  C – incentives + prescriber tools  D – pharmacists’ time + challenge prescribers + data  E – pharmacists’ time + challenge prescribers + data + audits  F – pharmacists’ time + challenge prescribers + guidance  G – pharmacists’ time + challenge prescribers + prescriber tools |
| - 1. Overall model construction | Combine the different types of intervention. | Evidence was triangulated from existing literature, the survey data analysis, an assessment of necessary conditions (step 4 above), and the initial round of fsQCA conducted within categories of interventions to inform which interventions should be used on overall fsQCA models (step 5 above, main text Table 3). Only combinations of interventions in the 8 hypothesised pathways were tested to avoid data-dredging (step 6 above).  The intermediate solution was used reflecting a balance between the most parsimonious solution (which includes the simplest set of ‘core conditions’) and the most complex solution (which includes all possible combinations of conditions)^9,10^.  This step identified which combinations of interventions were sufficient for the outcome: always when these combinations were present, the outcome was present. |
| - 1. Logical minimisation | To identify the simplest, least complex solutions consistent with the data. | Models were refined and minimised iteratively, starting with all the conditions in each category that were identified as necessary or sufficient, and removing conditions individually to see if the consistency and coverage scores changed. If the consistency score was either lower or the same when a condition was included in the model (compared to when it was not), it was removed. |
| - 1. Final solutions | Bring together the necessity analysis with the | The final model then consisted of the simplest combination of interventions for each model, with also the conditions identified as necessary from the necessity analysis:   - 1. Guidance (local guidance, NICE Infection Management, UKHSA guidance) and incentives (NOF, ICB-led)   2. Local guidance, Incentives (NOF) and data (ePACT2, Fingertips)   3. Local guidance, incentives (NOF, MO) and prescriber tools (Delayed prescribing, computerised decision support tools)   4. Local guidance, challenging prescribers and data (ePACT2, Fingertips)   5. Local guidance, data (ePACT2, Fingertips) and audits (ICB-led)   6. Guidance (Local guidance, UKHSA guidance), and challenging prescribers   7. Local guidance, challenging prescribers and prescriber tools (Delayed prescribing, computerised decision support tools) |
| - 1. Case analysis | Assess the impacts of different configurations of interventions at national level | Where a solution was deemed sufficient for an outcome to occur, ICBs which had implemented the combination of interventions in the solution were identified as a cluster of cases (implementation was defined as the calibrated fuzzy score for each condition in the solution being ≥0·7).  We the potential reductions in antibiotic prescribing at a national level were all ICBs to implement each configuration of interventions. The national annual difference in antibiotic items prescribed between implementing and non-implementing ICBs was calculated (difference in antibiotic prescribing per 1000 population x 1000 x 42 [number of ICBs] x 12 [months]) to identify the potential impact if all ICBs implemented each combination of interventions. |
| - 1. Contrarian case analysis | To identify any difference in solutions when the outcome did not occur^2^. | A contrarian case analysis was done by conducting a separate analysis where the outcome of interest was not meeting the antibiotic use target (i.e. the negation of the outcome). To do this, the same models were run, but with the outcome of interest set to 0 (i.e. not meeting target) rather than 1 (meeting target). |

### **Figure S1: Stages of fsQCA**

### **Table S3: Condition calibration**

| **Type of intervention** | **Survey response** | **Calibrated value** |
| --- | --- | --- |
| **Guidelines and toolkits** | Weekly | 1 |
|  | Monthly Every few months | 0.7 |
|  | Annually | 0.3 |
|  | Aware of but don't use Not heard of | 0 |
| **Workforce** | Any time protected | 1 |
|  | No time protected Unknown No role | 0 |
| **Governance** | Yes | 1 |
|  | No | 0 |
| **Monitoring & feedback – data** | Weekly | 1 |
|  | Monthly | 0.7 |
|  | Every few months | 0.5 |
|  | Annually | 0.3 |
|  | Aware of but don't use Not heard of | 0 |
| **Monitoring & feedback – audits** | Weekly  Monthly | 1 |
|  | Every few months  Annually | 0.7 |
|  | Aware of but don't use Not heard of | 0 |
| **Professional engagement & training** | 3 staff completed training 2 staff completed training | 1 |
|  | 1 staff completed training | 0.7 |
|  | 0 staff completed training Don't know | 0 |
| **Prescriber tools and diagnostics** | Daily | 1 |
|  | Weekly | 0.7 |
|  | Monthly | 0.5 |
|  | Every few months | 0.3 |
|  | Aware of but don't use Not heard of | 0 |
| **Policy, commissioning & incentives** | Very relevant (affects our work weekly or more) | 1 |
|  | Somewhat relevant (affects our work every few months) | 0.5 |
|  | Not relevant Not heard of | 0 |
| **Public awareness** | Weekly | 1 |
|  | Monthly | 0.7 |
|  | Every few months Annually | 0.3 |
|  | Aware of but don't use Not heard of | 0 |

### **Table S4: Outcome calibration**

| **Raw value**  (Antibiotic items per STAR-PU) | **Policy relevance** | **Calibrated** |
| --- | --- | --- |
| ≤0.871 | 2024 target (25% reduction from 2014) | 1 |
| ≤0.965 | Interim target | 0.5 |
| ≤1.161 | 1. baseline | 0 |

## D – Category fsQCA results

### **Table S5: Effective solutions identified in category-specific fsQCA (‘truth table’)**

| **Solution configurations** ** indicates AND; ~ indicates NOT* | **Coverage** | **Consistency** |
| --- | --- | --- |
| **GUIDANCE & TOOLKITS** |  |  |
| RoyalColleges*LocalGuidance*~FutureNHS*NICEInfectionManagement*NICEStewardship*~TARGET*~HowToGuides*UKHSAguidance | 0.20 | 0.84 |
| ~RoyalColleges*LocalGuidance*FutureNHS*NICEInfectionManagement*~NICEStewardship*TARGET*~HowToGuides*UKHSAguidance | 0.34 | 0.90 |
| RoyalColleges*~LocalGuidance*FutureNHS*NICEInfectionManagement*~NICEStewardship*TARGET*HowToGuides*UKHSAguidance | 0.13 | 0.80 |
| RoyalColleges*LocalGuidance*FutureNHS*NICEInfectionManagement*~NICEStewardship*TARGET*HowToGuides*UKHSAguidance | 0.32 | 0.81 |
| RoyalColleges*LocalGuidance*~FutureNHS*NICEInfectionManagement*NICEStewardship*TARGET*HowToGuides*UKHSAguidance | 0.18 | 0.82 |
| RoyalColleges*~LocalGuidance*FutureNHS*NICEInfectionManagement*NICEStewardship*TARGET*HowToGuides*UKHSAguidance | 0.13 | 0.80 |
| ~RoyalColleges*LocalGuidance*FutureNHS*NICEInfectionManagement*NICEStewardship*TARGET*HowToGuides*UKHSAguidance | 0.32 | 0.86 |
| **MONITORING & FEEDBACK** | | |
| ***(a) data only*** (5 most used datasets only): ePACT2*~GPSystem*Fingertips*OpenPrescribing*PrescQIPP | 0.32 | 0.91 |
| ***(b) audits only:*** No solutions identified | n/a | n/a |
| **POLICY, INCENTIVES & COMMISSIONING INTERVENTIONS** | | |
| NHSOversightFrameworks*ICBIncentives*~PrimaryCareNetworkDirectedEnhancedServices*~GPContract  *MedicinesOptimisationOpportunities*NationalActionPlan | 0.39 | 0.80 |
| **PROFESSIONAL ENGAGEMENT** | | |
| ~ShortEvents*~HalfDayEvents*~WholeDayEvents*~MultiDayEvents*~Courses | 0.12 | 0.81 |
| **PUBLIC AWARENESS INTERVENTIONS** | | |
| No solution identified | n/a | n/a |
| **PRESCRIBER TOOLS & DIAGNOSTICS** | | |
| ***(a) Prescriber tools only:*** ~ClinicalPredictionScores * ComputerisedDecisionSupportTools * DelayedPrescribing * DigitalPrompts * DigitalSafetyAlerts * TextsToPatients | 0.14 | 0.81 |
| ***(b) Diagnostics only:*** No solution identified | n/a | n/a |
| **WORKFORCE & GOVERNANCE** | | |
| ~HeadOfMedicinesOptimisation*~MedicinesOptimisationPharmacists*MedicinesOptimisationTechnicians* AttendICSsCommittee*~ChallengePrescribers*~AttendRegionalAMSmeetings | 0.08 | 1.0 |
| ~HeadOfMedicinesOptimisation*MedicinesOptimisationPharmacists*~MedicinesOptimisationTechnicians* ~AttendICSsCommittee*ChallengePrescribers* ~AttendRegionalAMSmeetings | 0.08 | 1.0 |

## E - Characteristics of ICBs

### **Table S6: Characteristics of ICBs that implemented different combinations of interventions**

|  |  | **Asthma prevalence (%)** | | | **Diabetes prevalence (%)** | | | **COPD prevalence (%)** | | | **Cancer prevalence (%)** | | | **CKD prevalence (%)** | | | **Deprivation**  **(IMD rank)** | | | **GPs per 100,000 population** | | |
| --- | --- | --- | --- | --- | --- | --- | --- | --- | --- | --- | --- | --- | --- | --- | --- | --- | --- | --- | --- | --- | --- | --- |
| **Model** | **Conditions** | Implementing ICBs | Non-implementing ICBs | Difference | Implementing ICBs | Non-implementing ICBs | Difference | Implementing ICBs | Non-implementing ICBs | Difference | Implementing ICBs | Non-implementing ICBs | Difference | Implementing ICBs | Non-implementing ICBs | Difference | Implementing ICBs | Non-implementing ICBs | Difference | Implementing ICBs | Non-implementing ICBs | Difference |
| A: Incentives + Guidance | NICE guidance*UKHSA guidance*NOF*ICB Incentives | 6.5 | 7.2 | -0.7 | 7.4 | 7.4 | 0.0 | 1.8 | 2.2 | -0.4 | 3.4 | 4.1 | -0.7 | 4.0 | 5.1 | -1.1 | 18.7 | 18.7 | 20.9 | -2.2 | 59.1 | 59.7 |
| B: Incentives + data | ePACT2*Fingertips* NOF | 6.4 | 7.1 | -0.7 | 7.4 | 7.5 | -0.1 | 1.8 | 2.1 | -0.3 | 3.3 | 4.0 | -0.7 | 3.0 | 4.9 | -1.9 | 19.2 | 19.2 | 20.9 | -1.7 | 59.2 | 60.0 |
| C: Incentives + prescriber tools | NOF*MO*CDSTs* Delayed prescribing | 6.7 | 6.8 | -0.1 | 7.4 | 7.5 | -0.1 | 1.9 | 1.9 | 0.0 | 3.4 | 3.7 | -0.3 | 4.0 | 4.5 | -0.5 | 22.6 | 22.6 | 19.0 | 3.6 | 60.3 | 58.9 |
| D: Challenge prescribers + data | ePACT2*Fingertips* Prescribers challenged | 6.5 | 6.9 | -0.4 | 7.2 | 7.6 | -0.4 | 1.8 | 2.0 | -0.2 | 3.6 | 3.7 | -0.1 | 4.2 | 4.5 | -0.3 | 19.7 | 19.7 | 18.6 | 1.1 | 59.8 | 58.9 |
| E: Data + audits | ePACT2*Fingertips* ICB audit | 6.2 | 7.1 | -0.9 | 7.3 | 7.6 | -0.3 | 1.7 | 2.1 | -0.4 | 3.2 | 4.0 | -0.8 | 3.6 | 4.9 | -1.3 | 23.6 | 23.6 | 16.4 | 7.2 | 57.9 | 59.9 |
| F: Challenge prescribers + guidance | Local guidance*UKHSA guidance*Prescribers challenged | 6.7 | 6.9 | -0.2 | 7.2 | 7.7 | -0.5 | 1.8 | 2.1 | -0.3 | 3.8 | 4.0 | -0.2 | 3.8 | 4.4 | -0.6 | 21.4 | 21.4 | 18.5 | 2.9 | 59.8 | 58.6 |
| G: Challenge prescribers + tools | CDSTs*Delayed prescribing* Prescribers challenged | 6.9 | 6.8 | 0.1 | 7.0 | 7.4 | -0.4 | 2.0 | 1.9 | 0.1 | 3.9 | 3.6 | 0.3 | 4.6 | 4.4 | 0.2 | 26.3 | 26.3 | 18.1 | 8.2 | 59.9 | 59.0 |

*ICB = integrated care board. NOF = NHS Oversight Framework. CDST = Computerised Decision Support Tools. MO = Medicines Optimisation Opportunities. NICE guidance refers to only Infection Management guidance (not NICE Stewardship guidance). COPD = chronic obstructive pulmonary disease. CKD = chronic kidney disease. For deprivation, ICBs were ranked by the average index of multiple deprivation scores of lower super output areas in their ICBs, with 1 being the ICBs most deprived*

## F – Contrarian case analysis

### **Table S7: Analysis results when the outcome of interest is not meeting the target**

|  | **Configurations** | | **Final model: fsQCA metrics** | | | **Contrarian case analysis: fsQCA metrics** | | | |
| --- | --- | --- | --- | --- | --- | --- | --- | --- | --- |
|  | **Summary**  **(in additional to local guidance)** | **Conditions** | **Coverage** | **Consistency** | **No. ICBs** | **Coverage** | **Consistency** | **No. ICBs** | **interpretation** |
| A | Incentives + guidance | Local guidance* NICE guidance* UKHSA guidance *NOF*ICB Incentives | 0·29 | 0·82 | 20 | 0.23 | 0.52 | - | Consistency threshold not met, therefore no solutions. No ICBs implemented this combination of interventions did not meet the target. |
| B | Incentives + data | Local guidance* ePACT2* Fingertips*NOF | 0·41 | 0·91 | 13 | 0.57 | 0.68 | - | Consistency threshold not met, therefore no solutions. No ICBs implemented this combination of interventions did not meet the target. |
| C | Incentives + prescriber tools | Local guidance* NOF*MO* CDSTs*Delayed prescribing | 0·28 | 0·89 | 9 | 0.41 | 0.91 | 3 | The solution included the absence of prescriber tools (CDSTs and delayed prescribing), but did include incentives (NOF, MO). Therefore, 3 ICBs did not meet the target whilst implementing incentives. This suggests that not all ICBs that implemented incentives met the target. |
| D | Challenge prescribers + data | Local guidance* ePACT2*Fingertips* Prescribers challenged | 0·34 | 0·87 | 9 | 0.37 | 0.63 | - | Consistency threshold not met, therefore no solutions. No ICBs implemented this combination of interventions did not meet the target. |
| E | Data + audits | Local guidance* ePACT2*Fingertips *ICB audit | 0·46 | 0·89 | 11 | 0.28 | 0.72 | - | Consistency threshold not met, therefore no solutions. No ICBs implemented this combination of interventions did not meet the target. |
| F | Challenge prescribers + guidance | Local guidance* UKHSA guidance* Prescribers challenged | 0·22 | 0·89 | 15 | 0.25 | 0.87 | 3 | The solution had the absence of UKHSA guidance and challenging prescribers, suggesting that only implementing local guidance occurred in the ICBs that did not meet the target. This aligns with what was already known from the necessity analysis (local guidance was needed for the outcome to occur but it was not sufficient). |
| G | Challenge prescribers + tools | Local guidance* CDST*Delayed prescribing* Prescribers challenged | 0·29 | 0·92 | 7 | 0.31 | 0.89 | 4 | 4 ICBs implemented different combinations of these interventions and did not meet the target. The one intervention in common was delayed prescribing. |

## G - References

1. Rihoux B, Ragin C. *Configurational comparative methods: Qualitative comparative analysis (QCA) and related techniques*. 2009.

2. Schneider CQ, Wagemann C. Standards of Good Practice in Qualitative Comparative Analysis (QCA) and Fuzzy-Sets. *Comparative Sociology* 2010; **9**: 397–418.

3. Mello PA. *Qualitative comparative analysis: An introduction to research design and application*. Washington DC: Georgetown University Press ; 2021.

4. Schneider CQ, Wagemann C. *Set-Theoretic Methods for the Social Sciences*. Cambridge University Press; 2012.

5. Avdagic S. When Are Concerted Reforms Feasible? Explaining the Emergence of Social Pacts in Western Europe. *http://dx.doi.org/101177/0010414009356178* 2010; **43**: 628–57. Available at: https://journals.sagepub.com/doi/abs/10.1177/0010414009356178. Accessed August 30, 2024.

6. Knowles R, Chandler C, O’Neill S, Sharland M, Mays N. A systematic review of national interventions and policies to optimize antibiotic use in healthcare settings in England. *Journal of Antimicrobial Chemotherapy* 2024; **79**: 1234–47. Available at: https://dx.doi.org/10.1093/jac/dkae061. Accessed September 6, 2024.

7. Eastmure E, Fraser A, Al-Haboubi M, *et al.* *Evaluation of the Implementation of the UK Antimicrobial Resistance (AMR) Strategy, 2013-2018*. London; 2019. Available at: http://piru.lshtm.ac.uk.

8. PIRU. *Logic model of the UK Antimicrobial Resistance (AMR) Strategy, 2013-2018*. 2019. Available at: https://piru.ac.uk/assets/files/App%201-Logic%20model%20of%20the%20UK%20AMR.pdf. Accessed September 6, 2024.

9. Pappas IO, Woodside AG. Fuzzy-set Qualitative Comparative Analysis (fsQCA): Guidelines for research practice in Information Systems and marketing. *Int J Inf Manage* 2021; **58**: 102310.

10. Ragin CC. *Redesigning social inquiry: Fuzzy sets and beyond*. Chicago: University of Chicago Press; 2008.
